# Insights Into the Enantioseparation of Polyhalogenated 4,4′‐Bipyridines With a Cellulose *Tris*(3,5‐Dimethylphenylcarbamate)‐Based Chiral Column by Using Supercritical Fluid Chromatography

**DOI:** 10.1002/elps.8156

**Published:** 2025-05-31

**Authors:** Emmanuelle Lipka, Roberto Dallocchio, Barbara Sechi, Mikheil Rukhaia, Giorgi Jibuti, Bezhan Chankvetadze, Victor Mamane, Paola Peluso

**Affiliations:** ^1^ Laboratoire de Chimie Analytique UMR 1167–UFR3S–Pharmacie Lille Cedex France; ^2^ Institute of Biomolecular Chemistry ICB CNR Li Punti Sassari Italy; ^3^ Institute of Applied Mathematics Tbilisi State University Tbilisi Georgia; ^4^ Institute of Physical and Analytical Chemistry School of Exact and Natural Sciences Tbilisi State University Tbilisi Georgia; ^5^ Institut de Chimie de Strasbourg UMR CNRS 7177 Equipe CLIC Strasbourg Cedex France

**Keywords:** bipyridines, carbon dioxide, enantioseparation, polysaccharide‐based chiral stationary phases, supercritical fluid chromatography

## Abstract

In the last decade, by integrating experimental and computational analyses, it was demonstrated that halogen bond (HaB) may contribute to binding and enantiorecognition mechanisms underlying the HPLC enantioseparation of halogenated chiral analytes by using cellulose *tris*(3,5‐dimethylphenylcarbamate) (CDMPC)‐based chiral columns and *n*‐hexane‐based mixtures as mobile phases. When used as a pivotal component of the mobile phase in supercritical fluid chromatography (SFC), carbon dioxide is often considered as an *n*‐hexane‐like nonpolar solvent because of its low dielectric constant and zero molecular dipole moment. On the other hand, carbon dioxide may also serve as hydrogen bond (HB) and HaB acceptor due to the presence of nonbonding electrons on the two oxygen atoms, interacting with analyte enantiomers, chiral selectors, and co‐solvents. On this basis, we report herein the results of a study aiming at evaluating the impact of using carbon dioxide in SFC in place of *n*‐hexane in HPLC on halogen‐dependent enantioseparations by using atropisomeric halogenated 4,4′‐bipyridines as analytes and Lux Cellulose‐1 as CDMPC‐based chiral column. The experimental investigation was complemented by a computational study performed using (a) quantum mechanics (QM) calculations to map and quantify noncovalent interactions possibly underlying the contact of the analytes with carbon dioxide and with the distinctive pendant groups of the CDMPC and (b) molecular dynamics (MD) simulations to visualize noncovalent interactions acting in the analyte **1**/CDMPC chromatographic system in different media. The use of MD simulations to model enantioseparations performed in carbon dioxide‐based media was not reported in the literature so far.

**Abbreviations**
2‐PrOH: propan‐2‐ol
CAM‐B3LYP: Coulomb‐attenuating method‐Becke's three‐parameter exchange functional with the Lee‐Yang‐Parr correlation functional
CDMPC: cellulose *tris*(3,5‐dimethylphenylcarbamate)
DFT: density functional theory
EEO: enantiomer elution order
*gd3*
: D3 version of Grimme's dispersion correction
HaB: halogen bond
HB: hydrogen bond
LanL2DZ: Los Alamos National Laboratory 2 double‐ζ
MD: molecular dynamics
MDMPC: methyl *tris*(3,5‐dimethylphenylcarbamate)
MeOH: methanol
QM: quantum mechanics
SFC: supercritical fluid chromatography

## 1 Introduction

Noncovalent interactions play a key role in separation and enantioseparation sciences [1]. In most chromatographic enantioseparations, a chiral selector linked to a stationary phase binds the enantiomers, recognizes the differences in their three‐dimensional structure, and makes them thermodynamically and kinetically differentiated through the formation of transient diastereomeric complexes. In liquid‐phase chromatography, this process occurs in the presence of the mobile phase, and enantioselective and nonenantioselective inter‐ and intramolecular [2, 3] noncovalent interactions may contribute to the binding and recognition processes resulting in analytical enantioseparation [1, 4]. All components of the enantioseparation system, chiral analyte, chiral selector, and mobile phase, are involved in noncovalent interactions, and the mobile phase may finely modulate the strength of these interactions. Furthermore, changing the nature of mobile phase may induce remarkable modifications of chiral selector conformation. This is particularly true for polysaccharide‐based selectors [5, 6]. Although hydrogen bond (HB) and π─π interactions have exerted a pivotal role in enantioseparation science [1, 7, 8], and HB‐based design strongly contributed to advance chirotechnology and method development in this field [1, 8, 9], over time other noncovalent interactions like halogen bond (HaB) [10, 11, 12] and chalcogen bond [13], along with dispersion and hydrophobic forces [14], attracted interest enlarging the scenario of the noncovalent forces potentially acting in chromatographic enantioseparations.

Chromatographic techniques proved to be very sensitive to detect weak noncovalent interactions compared to other analytical techniques [15] because they are based on reiterative adsorption‐desorption steps which amplify the effect of noncovalent interactions and, consequently, their detectability [1]. Furthermore, this feature makes chromatographic parameters suitable as benchmark data to validate computational tools and approaches used to explain and understand the enantioseparation processes at the molecular level [16, 17, 18, 19].

On the basis of the recommendations of the International Union of Pure and Applied Chemistry [20, 21], a HaB occurs when there is evidence of a net attractive interaction between an electrophilic region associated with a halogen atom in a molecular entity and a nucleophilic region in another, or the same, molecular entity. The strength of these directional noncovalent interactions depends on the nature of the halogen atom (HaB donor) as electron charge density acceptor and of the Lewis base (HaB acceptor) as electron charge density donor [21], and it increases in the order Cl < Br < I. On this basis, iodine is considered a powerful HaB donor due to its high polarizability and to the presence of a large region of electron density depletion on the elongation of the covalent bond involving the iodine atom. HaBs may be of van der Waals type, weak, strong, or very strong, with binding energies ranging between −0.01 and −100 kcal mol^−1^ [22]. The energy range of HaBs between interacting neutral molecular entities is often between −0.01 and −8.0 kcal mol^−1^. Although they were identified in the early 19th century [23], HaBs continue to attract great interest in crystal engineering and supramolecular chemistry and to be identified in many chemical and biological systems [23, 24, 25].

**FIGURE 1:**
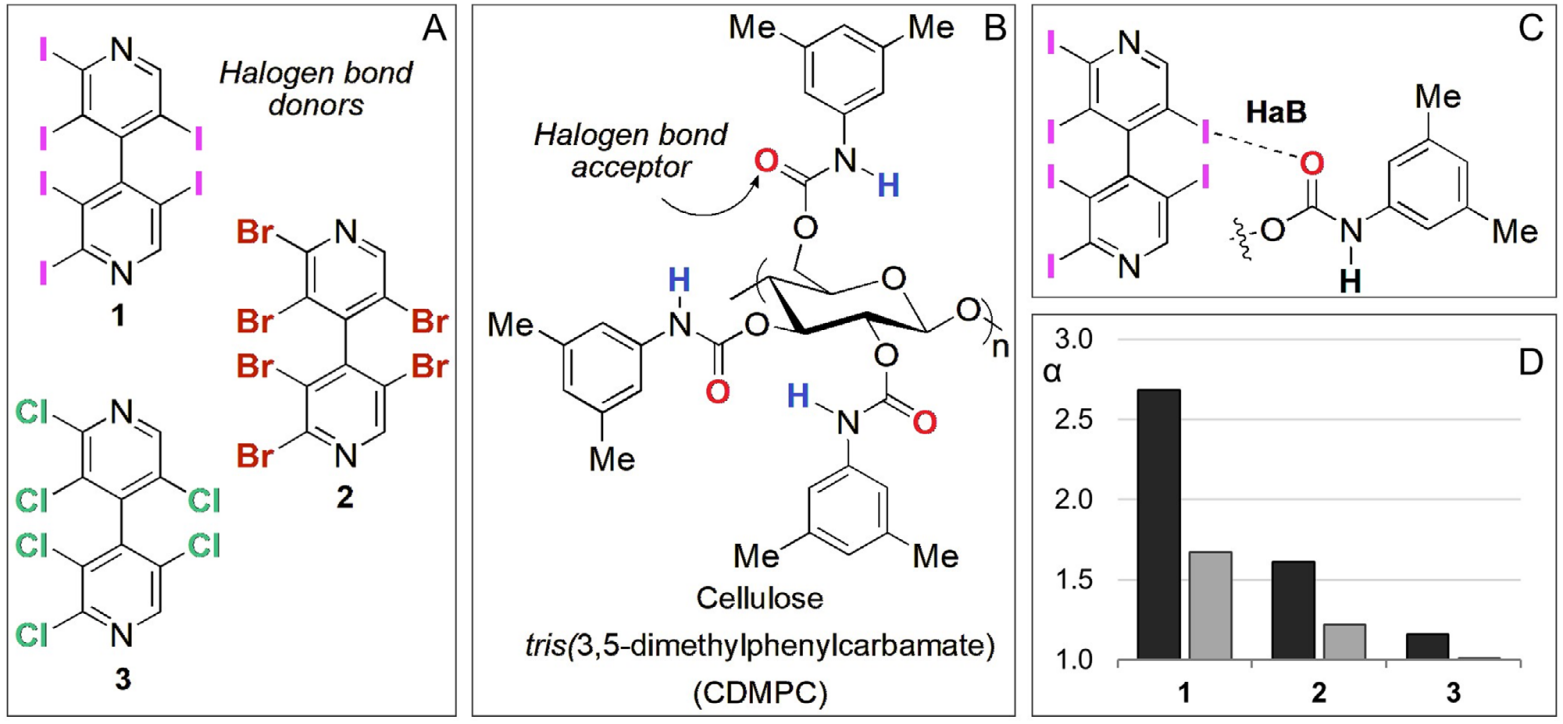
Structures of 2,2′,3,3′,5,5′‐hexahalogenated 4,4′‐bipyridines **1–3** (A) and of cellulose *tris*(3,5‐dimethylphenylcarbamate) (CDMPC) (B), description of halogen bond (HaB) between analyte **1** and the 3,5‐dimethylphenylcarbamate framework (C), and comparison of separation factors (*α*) obtained for 4,4′‐bipyridines **1–3** with Lux Cellulose‐1 (CDMPC) by using *n*‐hexane/2‐PrOH 90:10 v/v (black bars) and *n*‐hexane/2‐PrOH/MeOH 90:5:5 v/v/v (grey bars) as mobile phases (D) (Table S1 for numerical data [15]) (flow rate = 0.8 mL min^−1^, *T* = 25°C).

Studied in several fields of analytical chemistry for 20 years [9, 26, 27], HaBs were identified in liquid‐phase chromatography enantioseparation 10 years ago [28, 29]. By using *n*‐hexane/2‐PrOH 90:10 v/v as mobile phase and a cellulose *tris*(3,5‐dimethylphenylcarbamate) (CDMPC)‐based chiral column, the HPLC technique was very sensitive to detect HaBs as noncovalent interactions contributing to the enantioseparation of axially chiral 2,2′,3,3′,5,5′‐hexahalogenated 4,4′‐bipyridines like **1–3** (Figure 1A). In these cases, the enantioselectivity degree was clearly dependent on the electrophilic properties of chlorine, bromine, and iodine atoms featuring the analytes used as test probes and HaB donors, with the carbonyl oxygens of the CDMPC functioning as HaB acceptors on the other side (Figure 1B). As a result, HaBs were found to contribute to binding and enantiorecognition mechanisms underlying these enantioseparations (Figure 1C), observing that retention and selectivity values increased following the order Cl < Br < I (Figure 1D, black bars). It was also observed that retention and selectivity obtained for the enantioseparation of the halogenated 4,4′‐bipyridines were strongly reduced by introducing MeOH in the mobile phase (Figure 1D, grey bars). The effect was reasonably due to the competitive HBs between MeOH and the carbonyl oxygen atoms of the chiral selectors also functioning as HB acceptors, resulting in a reduced availability of these sites as HaB acceptors.

Nonpolar pressurized carbon dioxide, used as the basic component of the mobile phases in supercritical fluid chromatography (SFC), is often considered a *n*‐hexane‐like solvent with respect to its elution strength [30]. However, at molecular level, carbon dioxide is not really like *n*‐hexane because it has local dipoles, π electrons, and nonbonding electrons [31]. Furthermore, theoretical calculations and related experimental data confirmed that carbon dioxide can participate in conventional and nonconventional HBs [32] and form HaBs (O═C═O···X, X═Cl, Br, I) where it acts as HaB acceptor [33, 34].

On this basis, we report herein the results of an investigation on the SFC enantioseparation of polyhalogenated 4,4′‐bipyridines **1–3** on a CDMPC‐based chiral column. We aimed at evaluating the impact of changing *n*‐hexane to carbon dioxide as the major component of the mobile phase on the enantioseparation of **1–3** when SFC is used as chromatographic technique in place of HPLC. In other words, we used the SFC technique to evaluate (a) if retention and selectivity values obtained in SFC by using CO_2_/2‐PrOH mixtures as mobile phases could be correlated to the electrophilic properties of the halogen substituents increasing following the order Cl < Br < I, as already observed in HPLC by using *n*‐hexane/2‐PrOH 90:10 v/v as mobile phase, (b) the impact of changing *n*‐hexane to carbon dioxide on the enantioseparations of halogenated 4,4′‐bipyridines **1–3**, and (c) the impact of adding MeOH to the mobile phase on the SFC enantioseparations of 4,4′‐bipyridines **1–3**. Furthermore, quantum mechanics (QM) and molecular dynamics (MD) were used as computational techniques in a complementary manner to explain the experimental data at the molecular level. Indeed, QM calculations allowed for quantifying noncovalent interactions with higher accuracy compared to MD simulations. On the other hand, the MD technique is more suitable to describe explicit solvent effects and the dynamic features of the enantioseparation process.

## 2 Computationals

### 2.1 Quantum Mechanics

The 3D structures of the (*M*)‐ and (*P*)‐enantiomers of 4,4′‐bipyridines **1–3**, of carbon dioxide, of the methyl 3,5‐dimethylphenylcarbamate (MDMPC), and of related complexes were generated by using the build function and model kits and tools provided by GaussView 6.1 [35] for building and editing organic molecules. All structures used in this study were optimized at density functional theory (DFT) level in gas phase by using Gaussian 16W [36], with the hybrid exchange–correlation functional using the Coulomb‐attenuating method CAM‐B3LYP, the LanL2DZ (Los Alamos National Laboratory 2 double‐ζ) as effective core potential type basis set, and the D3 version of Grimme's dispersion correction (*gd3*). For each structure, the vibrational frequencies were also calculated to validate the optimization processes. For complexes of (*P*)‐**1–3** with carbon dioxide and with MDMPC, the basis set superposition error (BSSE) was corrected by the standard counterpoise method of Boys and Bernardi [37]. Interaction energies (Δ*E*_int_) are reported in kcal mol^−1^. Geometrical parameters, distances [Å], and angles (°) were measured by using the tools provided by GaussView 6.1 [35]. The strengths of HaBs and of HBs were evaluated by considering the percentage penetration degree of the van der Waals spheres of the interacting atoms [penetration parameter, *pp*% electrophile^…^nucleophile = 100 × {(*d*_El…Nu_)/(*r*_vdW_ El + *r*_vdW_ Nu) − 1}, where *d*_El…Nu_ is the interatomic distance between the interacting atoms with properties as electrophile and nucleophile, and *r*_vdW_ the corresponding van der Waals radii]. For the *r*_vdW_ of Br, Cl, H, I, N, and O, the values given by Bondi were considered [*r*_vdW_: 1.85 Å (Br), 1.75 Å (Cl), 1.20 Å (H), 1.98 Å (I), 1.55 Å (N), 1.52 Å (O)] [38].

### 2.2 Molecular Dynamics

The AMBER24 software [39] was used to carry out 100 ns MD simulations, and the Chimera software (UCSF, San Francisco, USA) was used for visualization and analysis of the MD trajectories [40]. MD details are reported in the Supporting Information section.

## 3 Materials and Methods

### 3.1 Chemicals and Chiral Stationary Phase

2,2′,3,3′,5,5′‐Hexahalogenated 4,4′‐bipyridines **1–3** were prepared and characterized as previously reported [41]. Lux Cellulose‐1 (CDMPC, 3 µm) (Phenomenex Inc., Torrance, CA, USA) was used as chiral column (250 × 4.6 mm). HPLC‐grade *n*‐hexane was purchased from Sigma‐Aldrich (Taufkirchen, Germany). Methanol (MeOH) and 2‐propanol (2‐PrOH) were purchased from Carlo‐Erba (Val‐de‐Reuil, France) and carbon dioxide (CO_2_, 99.995% purity) from Air Liquide (Loos, France).

### 3.2 Chromatographic Systems

An Agilent Technologies (Waldbronn, Germany) 1100 Series HPLC system (high‐pressure binary gradient system equipped with a diode array detector [DAD] operating at multiple wavelengths [220, 254, 280, and 360 nm], a programmable autosampler with a 20‐µL loop, and a thermostated column compartment) was employed. Data acquisition and analyses were carried out with Agilent Technologies ChemStation Version B.04.03 chromatographic data software.

The chromatographic system used for SFC analyses was a SFC‐PICLAB hybrid 10–20 apparatus (PIC Solution, Avignon, France) equipped with an autosampler comprising a 48‐vials plate (model Alias, Emmen, the Netherlands), three model 40 P pumps: two for CO_2_ and a third for the modifier (Knauer, Berlin, Germany), a column oven with a Valco ten‐position column selection valve, and a Valco six‐position solvent switching valve. The system was also composed of a Knauer Smartline 2600 DAD (Berlin, Germany). Detection wavelength was set at 254 nm. The system was controlled, and the data were acquired with the SFC‐PICLAB Analytic Online v.3.1.2 software (PIC Solution, Avignon, France). Retention times were average values of two replicate determinations. The data were processed by SFC New Data Manager V.1.8.0 software (PIC Solution, Avignon, France).

### 3.3 Chromatographic Conditions and Samples Preparation

For SFC analyses, the injected volume was 20 µL, the used flow rate was 1.5 mL min^−1^, and the mobile phase was always CO_2_‐modifier mixture with various percentages of MeOH or 2‐PrOH as modifiers. All analyses were run in isocratic mode. The column oven temperature was 40°C, and the outlet pressure was maintained at 120 bar for all experiments. The samples were prepared at 1 mg mL^−1^ in MeOH. The solutions were filtered through a 0.45 µm PTFE syringe‐filter (15 mm diameter) prior to use. HPLC analyses were performed in isocratic mode at 22°C. The flow rate was set at 0.8 mL min^−1^. The enantiomer elution order (EEO) for the enantioseparations of compounds **1–3** was determined by injecting pure enantiomers of known (*M*) or (*P*) absolute configuration [41].

## 4 Results and Discussion

### 4.1 SFC Enantioseparations of Halogenated 4,4′‐Bipyridines **1–3**

**FIGURE 2:**
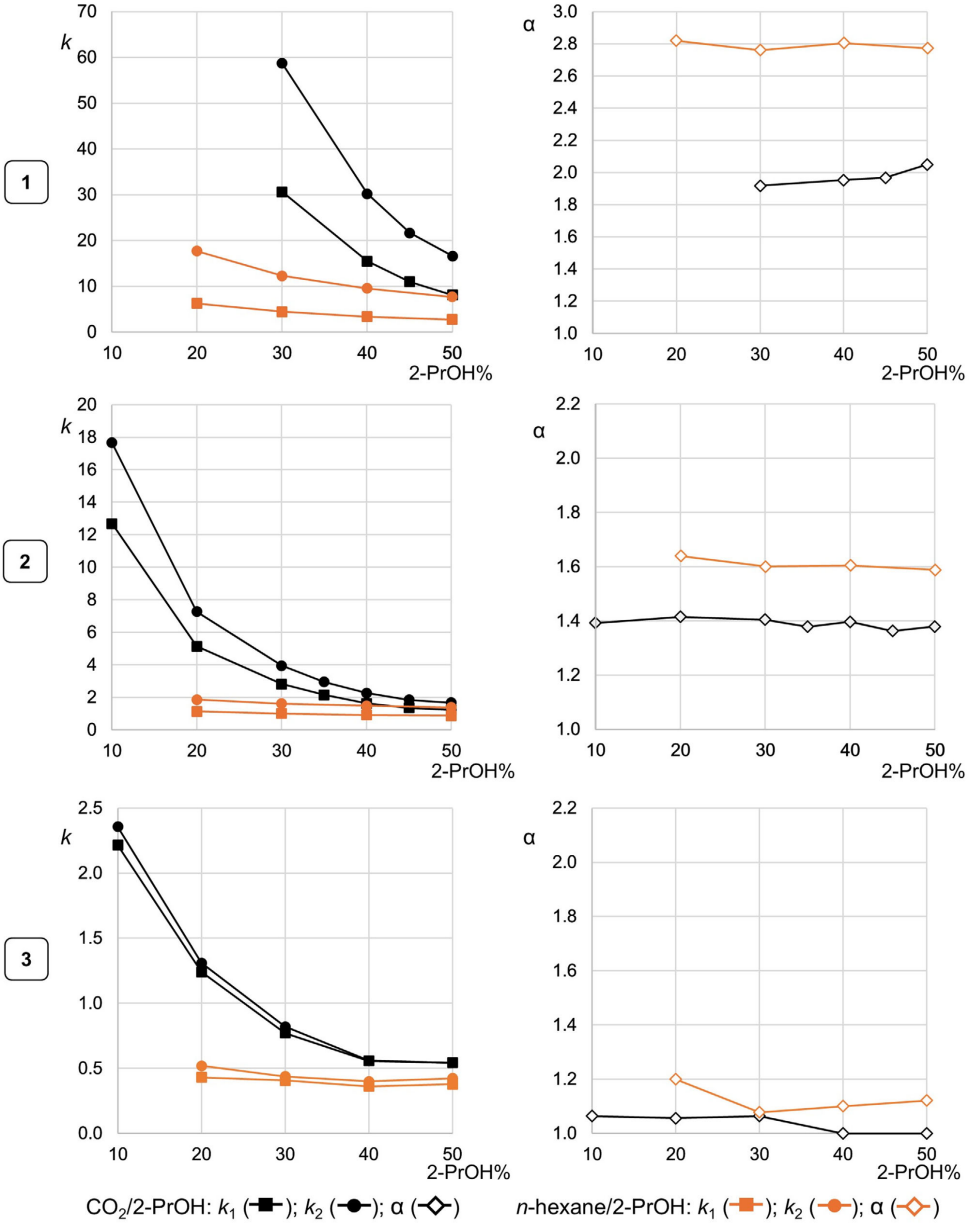
Dependence of retention (*k*) and separation (*α*) factors of *rac*‐**1**, *rac*‐**2**, and *rac*‐**3** on the content of 2‐PrOH in carbon dioxide/2‐PrOH (black lines) and *n*‐hexane/2‐PrOH (orange lines) mixtures used as mobile phases with Lux Cellulose‐1 as chiral column in SFC (flow rate = 1.5 mL min^−1^, *T* = 40°C) and HPLC (flow rate = 0.8 mL min^−1^, *T* = 22°C), respectively (for numerical data, Tables S2 and S3).

**FIGURE 3:**
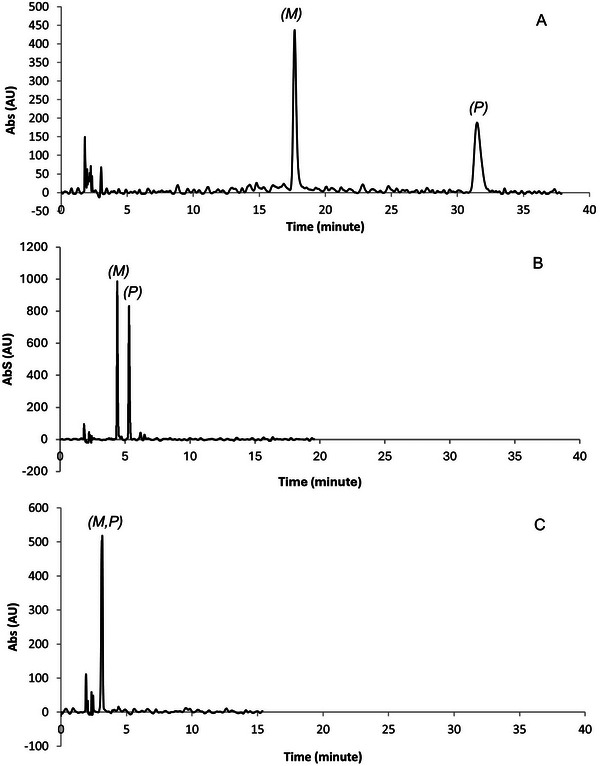
Separation and enantioseparation of *rac*‐**1** (A), *rac*‐**2** (B), and *rac*‐**3** (C) on Lux Cellulose‐1, carbon dioxide/2‐PrOH 50:50, flow rate = 1.5 mL min^−1^, *T* = 40°C, 150 bar, *λ* = 254 nm.

The SFC enantioseparation of compounds **1–3** was performed by using Lux Cellulose‐1 as chiral column with carbon dioxide/2‐PrOH mixtures as mobile phases with alcoholic additive content ranging from 50% to 10% (Figure 2, black lines). In agreement with the results obtained in the HPLC enantioseparation of **1–3** on the Lux Cellulose‐1 [29], in SFC both retention and selectivity also showed to be halogen‐dependent, increasing following the order Cl < Br < I. Thus, iodine‐based interactions seemed to contribute significantly to both binding and enantiorecognition mechanisms. This was evident even by using a mixture rich in alcoholic additive like carbon dioxide/2‐PrOH 50:50 v/v as mobile phase (Figure 3). Very high retention times were obtained for **1** by using carbon dioxide/2‐PrOH 70:30 v/v as mobile phase (*t*_1_ = 66.99 min; *t*_2_ = 126.54 min) compared to **2** and **3**. The elution of the iodinated analyte **1** was not obtained within 120 min by using mixtures with lower content of 2‐PrOH as mobile phases, like carbon dioxide/2‐PrOH 80:20 or 90:10 v/v.

Retention factors (*k*) increased significantly as the content of 2‐PrOH in the mobile phase decreased (and the carbon dioxide content increased) to an extent dependent on the distinctive halogen atoms. Indeed, by decreasing the content of 2‐PrOH in the mobile phase from 50% to 30%, the percentage increases for retention factors of the first and the second eluted enantiomers were 278–254, 130–135, and 42.6–51.8 for **1**, **2**, and **3**, respectively. Thus, increasing carbon dioxide content in the mobile phase had higher impact on retention of the iodinated **1** compared to 4,4′‐bipyridines **2** and **3**.

All compounds **1–3** showed (*M*)–(*P*) as EEO, suggesting that the same binding mechanism could be expected to underlie the affinity of each analyte to the chiral stationary phase, with difference related to the stereoelectronic properties of the distinctive analytes.

Separation factors (*α*) showed a negligible dependence on alcohol content in the mobile phase. However, a subtle difference could be observed between the selectivity trends of compounds **1** and **3**. Indeed, by increasing the content of 2‐PrOH from 30% to 50%, the separation factor of compound **1** also increased from 1.92 to 2.05, whereas an opposite trend could be observed for **3** with the separation factor slightly decreasing from 1.06 to 1.00.

Analytes **1–3** were also enantioseparated in HPLC by using the same CDMPC‐based chiral column and *n*‐hexane/2‐PrOH mixtures (20 ≤ 2‐PrOH% ≤ 50) as reference for comparison (Figure 2, orange lines). Higher retention factors were obtained in SFC compared to HPLC analysis. On this basis, carbon dioxide was shown to impact the affinity of the halogenated 4,4′‐bipyridines toward the polysaccharide‐based selector in a different way compared to *n*‐hexane and to an extent again dependent on the type of the distinctive halogen atoms. Indeed, by changing the analysis conditions from HPLC (*n*‐hexane/2‐PrOH 70:30, *T* = 22°C) to SFC (carbon dioxide/2‐PrOH 70:30, *T* = 40°C), the following percentage increases and decreases of retention factors of the first eluted enantiomers and selectivity values, respectively, could be observed: +586% and −30.4% for **1**, +178.2% and −12.5% for **2**, and +92.5% and −3.6% for **3**.

On the basis of the hypothesis that, in CDMPC‐based chiral stationary phases, the carbonyl oxygen atoms are acceptors for both HaB and HB donors, our results are consistent with the observation reported by West and co‐authors that the interaction ability of CDMPC toward HB donors is stronger in SFC compared to NPLC [42]. On the other hand, carbon dioxide/alcohol mixtures were reported to be heterogeneous, with alcohol molecules clustering around analytes [42], and the elution strength under heterogeneous conditions is higher than the strength expected for a homogeneous mixture. Furthermore, both carbon dioxide and alcohol as co‐solvent are adsorbed on the stationary phase, contributing to modifying its polarity and three‐dimensional structure [42, 43, 44].

The reduction of selectivity observed in SFC compared to HPLC was due to the different effects of changing *n*‐hexane to carbon dioxide on the retention of the second eluted enantiomers compared to the first eluted. For instance, considering mobile phases containing 30%, 40%, and 50% of 2‐PrOH, the average retention factor of the first eluted (*M*)‐enantiomer of **1** is five times higher in SFC than that in HPLC, whereas the average value for the second eluted enantiomer is 3.6 times higher. This effect is higher for **1** compared to **2** (2 times for *k*_1_ and 1.8 times for *k*_2_) and **3** (1.6 times for *k*_1_ and 1.5 times for *k*_2_), resulting in a higher decrease of selectivity by changing HPLC to SFC. At the molecular level, the same effect on both enantiomers could be expected if carbon dioxide would work as *n*‐hexane, functioning as a noninteracting solvent. On the contrary, an “enantioselective” effect of carbon dioxide may be envisaged if this medium exerts a different impact on the diastereomeric complexes between the halogenated analytes and the chiral selector. Furthermore, the different extents of the retention time increments dependent on halogen type (I > Br > Cl) could be related to the formation of HaB‐based O═C═O…halogen solvation clusters. On the other hand, the difference observed by changing *n*‐hexane to carbon dioxide may also originate from the interaction of carbon dioxide with the amidic N─H groups of the CDMPC affecting both selectivity and retention.

We also investigated the impact of changing 2‐PrOH to MeOH in carbon dioxide‐based mobile phases used in SFC (Table 1) by using content of MeOH ranging from 10% to 50%. Also in this case, retention times increased as the content of the alcoholic additive decreased, whereas the dependence of selectivity values on MeOH content was negligible. By using MeOH‐containing mobile phases, selectivity values decreased in all cases compared to those obtained with carbon dioxide/2‐PrOH mixtures to an extent following the order I > Br > Cl. This trend was also observed in HaB‐driven enantioseparations performed in HPLC [29].

**TABLE 1:** Retention times (*t*), retention factors (*k*), and separation factors (*α*) of 4,4′‐bipyridines **1–3** on Lux Cellulose‐1 [cellulose *tris*(3,5‐dimethylphenylcarbamate)] with carbon dioxide/2‐MeOH mixtures as mobile phases (flow rate = 1.5 mL min^−1^, *T* = 40°C).

4,4′‐Bipyridine	MeOH%	*t*_1_ (min)	*t*_2_ (min)	*k*_1_	*k*_2_	*α*
**1**	50	25.98	34.93	10.39	**14.32**	1.38
	45	38.36	54.11	15.53	22.32	1.44
	40	47.15	65.57	19.68	**27.76**	1.41
	35	64.33	89.18	26.14	**36.63**	1.40
	30	84.38	116.03	**30.37**	**42.13**	1.39
**2**	50	5.64	6.53	1.47	1.86	1.26
	45	6.42	7.48	1.77	2.22	1.26
	40	7.03	8.30	2.08	2.64	1.27
	35	8.16	9.79	2.44	3.13	1.28
	30	10.36	12.43	2.85	**3.62**	1.27
	20	15.76	19.47	7.25	9.19	1.27
	10	33.54	41.97	13.09	**16.63**	1.27
**3**	50	3.51	3.51	0.54	0.54	1
	40	3.87	3.87	0.70	0.70	1
	30	4.36	4.36	**0.62**	**0.62**	1
	20	5.49	5.49	1.87	1.87	1
	10	7.78	7.78	2.27	**2.27**	1

*Note*: Retention factors lower with MeOH‐containing mobile phases compared to 2‐PrOH‐containing media (Table S2) are reported in bold.

Differently, in most cases, retention factors increased by using MeOH as alcoholic component of the mobile phase likely due to the contribution of hydrophobic or dispersion forces to retention. Indeed, hydrophobic interactions are favored in MeOH more than in 2‐PrOH [45]. On the other hand, the polarizability of a chemical system may be correlated with its ability to exert dispersion‐based interactions given the higher mobility of the electrons [46]. Thus, the polarizability of MeOH being lower than that of 2‐PrOH, MeOH could exert lower competition for dispersion interactions than 2‐PrOH, enhancing selector‐analyte dispersion‐type interactions. Otherwise, in a few cases (highlighted in bold in Table 1), especially for the second eluted enantiomer of compound **1**, retention factors with the MeOH‐containing mobile phases decreased compared to mobile phases containing 2‐PrOH as cosolvent, likely due to the contribution of polar interactions that are weakened by using MeOH as csolvent.

All the subtle differences observed by changing *n*‐hexane to carbon dioxide in the mobile phases confirmed that carbon dioxide, as interacting solvent, behaves differently compared to the noninteracting *n*‐hexane and suggested that halogen‐dependent interactions occurred between carbon dioxide and analytes **1–3** in the SFC environment.

### 4.2 Computational Analysis

Halogen substituents can contribute to molecular recognition by playing multiple roles. Thus, possible formation of O═C═O^…^halogen noncovalent interactions required a theoretical confirmation. On this basis, QM methods applied in gas phase were used to identify and quantify O═C═O^…^I HaBs and other possible noncovalent interactions underlying the contact between analytes **1–3** and the methyl 3,5‐dimethylphenylcarbamate (MDMPC) framework as a model of the distinctive pendant group of the CDMPC chiral selector. Furthermore, MD simulations were also used to evaluate the impact of using carbon dioxide on the dynamic interactions between analyte **1** and a virtual nonamer of CDMPC by using explicit parametrization for solvent virtualization. In this regard, it is worth mentioning that MD simulations of SFC enantioseparations treating carbon dioxide explicitly were not reported so far.

In a previous study, the electrophilic properties of the halogen atoms featuring analytes **1–3** were evaluated as a prerequisite for HaB formation and quantified through electrostatic potential analysis, confirming an electrophilic character increasing following the order Cl < Br < I [15, 26].

In all computations, no energy constraint was applied to the interacting partners to force them to be in a specific position.

#### 4.2.1 QM Quantification of Noncovalent Interactions

To map possible noncovalent interactions between analytes **1–3** and carbon dioxide as interacting counterparts, we computed DFT‐optimized structures of nine carbon dioxide/analyte complexes (Figure S1) starting from nine input structures built by manually docking one molecule of carbon dioxide in proximity to each halogen atom at the 2‐, 3‐, and 5‐positions of the 4,4′‐bipyridyl scaffold. Given the symmetry of the analyte substitution pattern, 2′‐, 3′‐, and 5′‐positions were not considered. This approach aimed at providing just an estimate of the type and strength of noncovalent interactions involved in complex formation. On the other hand, cooperative or anti‐cooperative noncovalent effects may act in a real chromatographic system at the molecular level due to possible simultaneous interactions of the analyte with multiple solvent molecules and pendant groups and with the polymer backbone.

To obtain reliable interaction energies and noncovalent interaction maps, and considering the presence of the highly polarizable iodine atoms featuring analyte **1**, we used a computational method based on (a) the CAM‐B3LYP functional which includes a correction for long‐range interactions like dispersion forces, (b) D3 version of Grimme's dispersion correction (*gd3*), (c) the LanL2DZ as an effective core potential type basis set to replace the core electrons with an effective potential and, consequently, to avoid the use of the core basis functions which would have increased computational time, and (d) the standard counterpoise method of Boys and Bernardi to correct the BSSE which would have affected interaction energies.

In Table 2, the noncovalent interactions underlying complex formation are reported. HaBs were observed in the carbon dioxide/**1** complexes, exclusively, with penetration degree of the van der Waals spheres ranging from −6.17% to −7.69%.

**TABLE 2:** Noncovalent interactions in density functional theory (DFT) optimized complexes between 4,4′‐bipyridines **1–3** and CO_2_ (color legend: carbon, gray; hydrogen, pale grey; nitrogen, blue; oxygen, red) (three‐dimensional structures of the optimized complexes are reported in Figure S1).

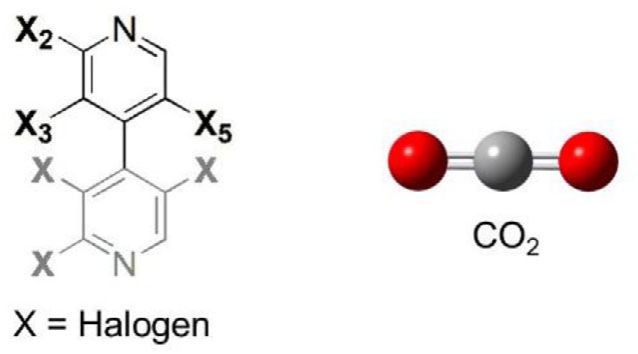		
Halogen	*E*_int_ (kcal mol^−1^)	Noncovalent interaction^a^
2‐I (**1**)	−1.81	2‐I…O═C═O (*d* = 3.284 Å, *pp*% = −6.17%)
2‐Br (**2**)	−4.17	N_pyr_…CO_2_ (*d* = 2.889 Å)
2‐Cl (**3**)	−3.39	π‐hole_pyr_^…^O═C═O (*d* = 3.178 Å)
3‐I (**1**)	−2.28	3‐I^…^O═C═O (*d* = 3.231 Å, *pp*% = −7.69%)
3‐Br (**2**)	−4.17	N_pyr_^…^CO_2_ (*d* = 2.889 Å)
3‐Cl (**3**)	−3.46	π‐hole_pyr_^…^O═C═O (*d* = 3.174 Å)
5‐I (**1**)	−2.20	5‐I^…^O═C═O (*d* = 3.243 Å, *pp*% = −7.34%)
5‐Br (**2**)	−2.78	π‐hole_pyr_^…^O═C═O (*d* = 3.062 Å)
5‐Cl (**3**)	−3.32	π‐hole_pyr_^…^O═C═O (*d* = 3.061 Å)

^a^ *pp*% = 100 × {(*d*_Ha⋅⋅⋅O_)/(*r*_vdW_ Ha + *r*_vdW_ O) − 1}), where *d*_Ha…O_ is the interatomic distance between Ha and O atoms and *r*_vdW_ the corresponding van der Waals radii.

For the complexes of carbon dioxide with **2** and **3**, other types of noncovalent interactions could be observed with no direct involvement of bromine or chlorine atoms as electrophiles. For these two analytes, the oxygen atoms of carbon dioxide are rather involved in noncovalent interactions involving the electron‐poor pyridyl π‐cloud as electrophile. These results may suggest that the formation of O═C═O^…^halogen HaBs may really be responsible for the high elution times and the lower selectivity observed for analyte **1** in SFC compared to HPLC, affecting the enantioseparation process in multiple possible ways. For instance, carbon dioxide/analyte clusters based on HaBs could be formed, which may slow the elution of the analyte along the surface of the CDMPC‐based chiral stationary phase. Moreover, in carbon dioxide‐based mobile phases, clusters could be also formed around the analyte/chiral selector complexes, which may reduce the stereoelectronic capability of the two enantiomers to be differentiated compared to the noninteracting *n*‐hexane used as the main component of the mobile phase in HPLC. On the other hand, the formation of HBs between carbon dioxide and the amidic hydrogen of the CDMPC (O═C═O…H─N<) may enhance the HB acceptor ability of the chiral selector and, consequently, the strength of noncovalent interactions involving the carbonyl oxygen atoms as HB or HaB acceptors and, in turn, the affinity of the analytes toward the stationary phase.

We also computed DFT‐optimized structures of three sets of complexes between each analyte and the MDMPC, with input structures built as follows: (*set 1*) The carbonyl group of MDMPC was located in proximity to each halogen atom at the 2‐, 3‐, and 5‐positions (Figure S2); (*set 2*) the amidic N─H group of MDMPC was located in proximity to the nucleophilic belt of each halogen atom at the 2‐, 3‐, and 5‐positions (Figure S3); (*set 3*) the amidic N─H group was located in proximity to the pyridyl nitrogen of each analyte as HB acceptor (Figure S4). Multiple noncovalent interactions were found to underlie most analyte/MDMPC complexes (Table 3).

It is worth noting that in the real system, noncovalent interactions may be affected by the stereoelectronic constraints acting on the analyte in the polymeric groove and by solvation effects. For instance, possible HBs involving the halogen atoms of analytes **1–3** as HB acceptors are expected to be rather weak and disfavored due to the low accessibility of the nucleophilic belt of these halogen atoms for a possible electrophile as HB donor (Figure S5). The same consideration may concern possible π‐π interactions involving the pyridyl rings of the analytes. Despite that, the QM computations could provide an estimate of the noncovalent interactions acting in the chromatographic system under investigation. Thirty‐five noncovalent interactions were found in the 21 optimized complexes (n. 7 for each analyte) with the following frequency: HaBs (n. 10) > π–π stacking interactions (n. 9) > HBs with *pp*% < 0 (n. 7) and weak HBs with *pp*% > 0 (n. 7) > π‐hole bonds (n. 2). This frequency agreed with the results of MD simulations reported previously for the complexes 4,4′‐bipyridine/CDMPC [15] where HaBs, π–π stacking interactions, and HBs were clearly detected. Furthermore, experimental data had evidenced the key contribution of the carbonyl oxygen atoms of the carbamate pendant groups compared to the amidic hydrogen atoms for halogen‐dependent enantioseparations performed by using CDMPC‐based chiral columns [15]. The average energy computed for the complexes of each distinctive analytes decreased following the order I > Cl > Br. The HaB (I…O═C< and I…ArMe_2_) was the most frequent interaction identified in the seven iodine‐containing complexes.

The most stable complexes involved the pyridyl nitrogen (N_pyr_…HN HB, −13.11 kcal mol^−1^) and the iodine at the 3‐ and 5‐positions (−12.69 and −12.56 kcal mol^−1^, respectively). Previous studies demonstrated that interactions involving sites located at the 3,3′,5,5′‐positions, close to the chiral axis, contribute significantly to the enantiorecognition [47]. On the contrary, complexes underlain by I^…^H─N< HBs, thus involving the amidic hydrogen of MDMPC and the electron‐rich belt of the iodine atoms as HB acceptors, showed lower interaction energies. Otherwise, π–π stacking interactions were found to be the most frequent interactions in the seven chlorine‐containing complexes, and the most stable complexes of analyte **3** were underlain by ArMe_2_…pyridyl π–π stacking. For the complexes involving analyte **2**, a widespread distribution between the five types of noncovalent interactions was obtained, with a prevalence of HaBs and HBs over π–π stacking interactions.

These QM calculations clearly showed that the iodine atoms of analyte **1** can form HaBs with both carbon dioxide and the pendant group of the CDMPC. In this regard, more negative average energies were computed for the MDMPC/**1** complexes (−10.37 kcal mol^−1^) compared to those computed for the MDMPC/**2** (−9.03 kcal mol^−1^) and MDMPC/**3** (−10.04 kcal mol^−1^) complexes. Thus, these observations confirmed, on QM bases, the contribution of HaBs in analyte‐selector contact, the higher affinity of analyte **1** toward the CDMPC, and the interactive properties of carbon dioxide toward analytes **1–3**.

**TABLE 3:** Noncovalent interactions in density functional theory (DFT) optimized complexes between 4,4′‐bipyridines **1–3** and methyl 3,5‐dimethylphenylcarbamate (MDMPC) (color legend: carbon, grey; hydrogen, pale grey; nitrogen, blue; oxygen, red) (three‐dimensional structures of the optimized complexes are reported in Figures S2–S4).

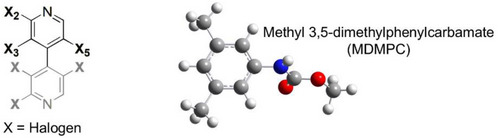			
Halogen	MDMPC (C═O or N─H)	*E*_int_ (kcal mol^−1^)	Noncovalent interaction^a^
*Set 1*			
2‐I (**1**)	C═O	−8.09	2‐I…O═C< (*d* = 3.285 Å, *pp*% = −6.14%) 3‐I…ArMe_2_ (*d* = 3.636 Å)
2‐Br (**2**)	C═O	−6.34	2‐Br…O═C< (*d* = 3.297 Å, *pp*% = −2.17%) 3‐Br…ArMe_2_ (*d* = 3.659 Å)
2‐Cl (**3**)	C═O	−10.37	2‐Cl…O═C< (*d* = 3.022 Å, *pp*% = −7.58%) ArMe_2_…Pyr π–π stacking
3‐I (**1**)	C═O	−12.69	3‐I·…O═C< (*d* = 3.055 Å, *pp*% = −12.71%) N_pyr_…HC‐Ar (*d* = 2.821 Å, *pp*% > 0) ArMe_2_…Pyr π–π stacking
3‐Br (**2**)	C═O	−11.37	3‐Br…O═C< (*d* = 3.031 Å, *pp*% = −10.06%) ArMe_2_…Pyr π–π stacking
3‐Cl (**3**)	C═O	−11.47	ArMe_2_…Pyr π–π stacking
5‐I (**1**)	C═O	−12.56	5‐I…O═C< (*d* = 3.172 Å, *pp*% = −9.37%) ArMe_2_…Pyr π–π stacking
5‐Br (**2**)	C═O	−8.94	π‐hole_pyr_…O═C< (*d* = 3.004 Å) N_pyr_…HC‐Ar (*d* = 2.560 Å, *pp*% = −6.91%)
5‐Cl (**3**)	C═O	−9.75	π‐hole_pyr_…O═C< (*d* = 3.061 Å)
*Set 2*			
2‐I (**1**)	N─H	−7.51	2‐I…H─N< (*d* = 3.482 Å, *pp*% > 0)
2‐Br (**2**)	N─H	−9.99	—
2‐Cl (**3**)	N─H	−10.27	2‐Cl…H─N< (*d* = 3.023 Å, *pp*% > 0) 5‐Cl…O═C< (*d* = 3.045 Å, *pp*% = −6.88%) ArMe_2_…Pyr π–π stacking
3‐I (**1**)	N─H	−9.26	3‐I…H─N< (*d* = 3.280 Å, *pp*% > 0) N_pyr_…HC‐Ar (*d* = 2.645 Å, *pp*% = −2.80%)
3‐Br (**2**)	N─H	−7.71	3‐Br…H─N< (*d* = 3.286 Å, *pp*% > 0) N_pyr_…HC‐Ar (*d* = 2.673 Å, *pp*% = −2.80%)
3‐Cl (**3**)	N─H	−8.89	3‐Cl…O═C< (*d* = 3.119 Å, *pp*% = −4.60%) N_pyr_…HC‐Ar (*d* = 2.784 Å, *pp*% > 0)
5‐I (**1**)	N─H	−9.36	ArMe_2_…Pyr π–π stacking
5‐Br (**2**)	N─H	−9.70	5‐Br…H─N< (*d* = 2.912 Å, *pp*% = −4.52%) N_pyr_…HC‐Ar (*d* = 2.776 Å, *pp*% > 0) ArMe_2_…Pyr π–π stacking
5‐Cl (**3**)	N─H	−10.93	ArMe_2_…Pyr π–π stacking
*Set 3*			
N_pyr_ (**1**)	N─H	−13.11	N_pyr_…H─N< (*d* = 2.474 Å, *pp*% = −10%)
N_pyr_ (**2**)	N─H	−9.16	N_pyr_…H─N< (*d* = 2.014 Å, *pp*% = −26.76%)
N_pyr_ (**3**)	N─H	−8.57	N_pyr_…H─N< (*d* = 2.060 Å, *pp*% = −25.09%)

^a^ *pp*% = 100 × {(*d*_El…Nu_)/(*r*_vdW_ El + *r*_vdW_ Nu) − 1}), where *d*_El…Nu_ is the interatomic distance between the interacting atoms with properties as electrophile and nucleophile and *r*_vdW_ the corresponding van der Waals radii.

#### 4.2.2 MD Simulations

The enantioseparations of **1** on Lux Cellulose‐1 with *n*‐hexane/2‐PrOH and carbon dioxide/2‐PrOH 70:30 v/v as mobile phases were considered, modeled by MD, and the computed results compared with the benchmark experimental data. These MD simulations were performed with the main aim to model the CDMPC in carbon dioxide‐based media, a type of simulations not reported so far in the field of enantioselective chromatography. Furthermore, our interest was also to confirm if and how HaBs participate in binding and enantioselective recognition in a dynamic perspective in the domain of molecular mechanics and to explore the noncovalent interaction pattern underlying the contact of CDMPC with analyte **1**. The 100 ns MD simulations in the AMBER force field [48] were performed by using a CDMPC nonamer as a virtual model of the polysaccharide‐based selector, DFT‐optimized structures computed for the (*M*)‐ and (*P*)‐enantiomers of **1**, and explicit virtual solvent mixtures in accord with the experimental conditions used in the chromatographic analyses. The explicit σ‐hole (ESH) parametrization [49, 50] was used to model the electrophilic electron charge density depletion on the iodine atoms [51] (see the Supporting Information section for details and Table S4).

**FIGURE 4:**
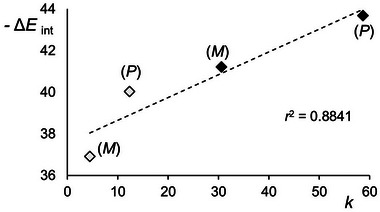
Correlation of the interaction energies (Δ*E*_int_, kcal mol^−1^) between the enantiomers of analyte **1** and a nonamer of CDMPC, computed by MD [solvent box: *n*‐hexane/2‐PrOH 70:30 (◊); carbon dioxide/2‐PrOH 70:30 (⧫)], with the experimental *k* of the first and the second eluted enantiomers of analyte **1** [Lux Cellulose‐1: *n*‐hexane/2‐PrOH 70:30, *T* = 22°C, 0.8 mL min^−1^ (◊); carbon dioxide/2‐PrOH 70:30, *T* = 40°C, 1.5 mL min^−1^ (⧫)].

In Figure 4, the correlation between the total interaction energies computed for the (*M*)‐**1**/CDMPC and (*P*)‐**1**/CDMPC complexes with the experimental retention factors is reported. Although the limitation of not considering the differences between the two environments (carbon dioxide vs. *n*‐hexane) related to the entropic effects, a satisfactory linear trendline (*r* ^2^ = 0.8841) was obtained.

The reported energies are mean values that were calculated from 5000 complexes obtained by snapshots taken every 20 ps from the 100 ns MD trajectories. The interaction energy (*E*_int_) between enantiomer and selector was calculated based on the energies of the selector–enantiomer complex, the selector, and the enantiomer (𝐸_int_ = 𝐸_total_ − 𝐸_enantiomer_ − 𝐸_polysaccharide_ _–_ _based selector_).

The computed EEOs [(*M*)–(*P*)] were in accordance with the experimental elution order, and more negative interaction energy values for the analyte‐CDMPC complexes were computed by using the carbon dioxide‐containing virtual mixture (Δ*E*_int_ (*M*) = −41.22 ± 3.96 kcal mol^−1^; Δ*E*_int_ (*P*) = −43.68 ± 5.59 kcal mol^−1^) compared to the *n*‐hexane‐based solvent (Δ*E*_int_ (*M*) = −36.92 ± 5.96 kcal mol^−1^; Δ*E*_int_ (*P*) = −40.04 ± 7.47 kcal mol^−1^).

In Tables S5–S8, lifetimes of HBs and HaBs extracted from the simulated MD trajectories of the (*M*)‐ and (*P*)‐**1** complexes with CDMPC in *n*‐hexane‐ and carbon dioxide‐based media are listed and compared. Furthermore, intramolecular π–π interactions within the CDMPC as well as I^…^π_ArMe2_ analyte‐CDMPC HaBs were also observed. By examining the noncovalent interaction patterns occurring in the two selected media, the following remarks emerged:

1. In the simulations performed with carbon dioxide/2‐PrOH 70:30, HBs between 2‐PrOH molecules (0.02 ≤ lifetime (ns) ≤ 8) stronger than those between carbon dioxide and 2‐PrOH (0.02 ≤ lifetime (ns) ≤ 0.2) were observed, confirming the heterogeneity of the carbon dioxide‐based medium.
2. In all simulations, in accordance with the pivotal feature of the highly ordered structure of polysaccharide carbamate‐based chiral selectors [5, 9], intramolecular >C═O…H─N< HBs could be observed during 100 ns MD with lifetime ranging from 63 to 0.02 ns.
3. In *n*‐hexane/2‐PrOH 70:30, CDMPC was solvated by 2‐PrOH molecules through HBs where solvent molecules acted as HB donors and acceptors interacting with carbonyl oxygen and the amidic hydrogen atoms, with lifetime ranging from 34 to 0.02 ns. In carbon dioxide/2‐PrOH 70:30, stronger HBs between 2‐PrOH and CDMPC were found with lifetimes ranging from 77 to 0.02 ns, but carbon dioxide molecules also solvated the polymer through several weaker HBs featuring lifetimes ranging from 2 to 0.02 ns. This confirmed that both carbon dioxide and 2‐PrOH adsorb on the stationary phase.
4. In *n*‐hexane/2‐PrOH 70:30, analyte enantiomers were solvated by 2‐PrOH molecules through weaker HBs between the hydroxyl hydrogen atoms of 2‐PrOH and the pyridyl nitrogen of the analyte (0.02 ≤ lifetime (ns) ≤ 3), and through stronger HaBs between the hydroxyl oxygen atoms of 2‐PrOH and the iodine atoms of the analyte (0.02 ≤ lifetime (ns) ≤ 10). In carbon dioxide/2‐PrOH 70:30, carbon dioxide molecules also solvated analyte enantiomers through several weaker HaBs featuring lifetimes ranging from 0.02 to 0.4 ns, whereas HBs (0.02 ≤ lifetime (ns) ≤ 2) and stronger HaBs (0.02 ≤ lifetime (ns) ≤ 35) between 2‐PrOH and the analyte could be also observed, confirming the presence of alcohol molecules clustering the analyte.
5. In all cases, the interaction between (*M*)‐ and (*P*)‐**1** enantiomers and the CDMPC was dominated by HaBs between iodine and the carbonyl oxygen atoms of the polymer with the number of observed HaBs and related penetration degrees in agreement with the experimental EEO [(*M*)–(*P*)] (Table 4). Furthermore, HBs between the pyridyl nitrogen atom of the analyte and the amidic hydrogen atom of the CDMPC also contribute to analyte‐chiral selector contact for the (*P*)‐enantiomers (0.6 ns) in *n*‐hexane/2‐PrOH 70:30 and for both (*M*)‐ and (*P*)‐enantiomers (0.1 and 9 ns, respectively) in carbon dioxide/2‐PrOH 70:30.
6. Interestingly, MD simulations performed by using carbon dioxide as medium showed strong intramolecular HBs within the CDMPC (0.02 ≤ lifetime (ns) ≤ 74) and intermolecular analyte‐CDMPC HaBs (0.4 ≤ lifetime (ns) ≤ 91) (Table 4 and Tables S9 and S10).
7. In all simulations involving carbon dioxide, solvent molecules were observed to preferentially coordinate the (*M*)‐enantiomer (0.02 ≤ lifetime (ns) ≤ 0.4) of the analyte compared to the most retained (*P*)‐enantiomer (0.02 ≤ lifetime (ns) ≤ 0.3). This preference could explain the higher retention increase observed experimentally for the first eluted enantiomer by changing HPLC to SFC conditions compared to the second eluted enantiomer. This behavior may be considered rather logical. Indeed, the second eluted (*P*)‐enantiomer exerts stronger interactions with the CDMPC. This reduces the electrophilic character of the other noninteracting iodine atoms and, consequently, their capability to coordinate carbon dioxide. The opposite occurs for the first eluted (*M*)‐enantiomer, which exerts less strong interactions with the CDMPC.

**TABLE 4:** Lifetime, penetration parameter%, and site location of the halogen bonds (HaBs) observed between (*M*)‐ and (*P*)‐enantiomers of **1** and the cellulose *tris*(3,5‐dimethylphenylcarbamate) (CDMPC) in 100 ns molecular dynamics (MDs) performed by using *n*‐hexane/2‐PrOH 70:30, CO_2_/2‐PrOH 70:30, and CO_2_ as virtual media. For the carbonyl groups of the CDMPC, the position of the corresponding pendant group in the glucopyranose unit is reported (2, 3, or 6).

Enantiomer	Lifetime (ns)	*pp*%^a^	Distance (Å)	Site location
*n‐*Hexane/2‐PrOH				
(*M*)‐**1**	78	−11.40	3.10	2′‐I…O═C< (6)
	51	−12.60	3.06	5′‐I…O═C< (6)
	43	−10.90	3.12	5‐I…O═C< (2)
(*P*)‐**1**	70	−12.60	3.06	5‐I…O═C< (6)
	33	−10.90	3.12	2′‐I…O═C< (6)
	11	−11.71	3.09	3′‐I…O═C< (6)
	8	−6.60	3.27	5′‐I…O═C< (3)
	6.3	−13.43	3.03	2‐I…O═C< (6)
	1.3	−1.10	3.46	2‐I…O═C< (2)
CO_2_/2‐PrOH				
(*M*)‐**1**	99	−12.00	3.08	2′‐I…O═C< (6)
	55	−13.43	3.03	5′‐I…O═C< (6)
	2.8	−7.43	3.24	5‐I…O═C< (2)
(*P*)‐**1**	73	−12.00	3.08	5‐I…O═C< (6)
	46	−11.71	3.09	3′‐I…O═C< (6)
	18	−11.41	3.11	2‐I…O═C< (6)
CO_2_				
(*M*)‐**1**	83	−12.86	3.05	2‐I…O═C< (6)
	66	7.71	3.23	5‐I…O═C< (6)
	15	−12.29	3.07	5′‐I…O═C< (6)
(*P*)‐**1**	91	−12.57	3.06	5‐I…O═C< (6)
	85	−12.57	3.06	3′‐I…O═C< (2)
	5.8	−10.00	3.15	2‐I…O═C< (6)
	5	−12.00	3.08	5′‐I…O═C< (6)
	0.4	−8.57	3.20	2‐I…O═C< (2)

^a^ *pp*% = 100 × {(*d*_Ha⋅⋅⋅O_)/(*r*_vdW_ Ha + *r*_vdW_ O) − 1}), where *d*_Ha…O_ is the interatomic distance between Ha and O atoms and *r*_vdW_ the corresponding van der Waals radii.

Representative snapshots of (*M*)‐ and (*P*)‐**1**/CDMPC complexes computed by MD and some related noncovalent interactions are reported in Tables S11 and S12 and Figure S6.

## 5 Concluding Remarks

The enantioseparation of three chiral 2,2′,3,3′,5,5′‐hexahalogenated 4,4′‐bipyridines was investigated by using a CDMPC‐based chiral column under SFC conditions. Each analyte features distinctive halogen atoms, iodine, bromine, and chlorine, as substituents of the 4,4′‐bipyridyl moiety. Given that polarizability and electrophilic properties of the series increase following the order Cl < Br < I, the three analytes were selected as test probes to explore the possibility that HaBs may contribute to binding and enantioselective recognition mechanisms in carbon dioxide media. The analyses were also performed by using the HPLC technique with *n*‐hexane‐based mobile phases as reference for comparison. Indeed, previous studies demonstrated that the enantioseparation of halogenated 4,4′‐bipyridines is controlled and modulated by HaBs in HPLC conditions, to a degree dependent on the electrophilic properties of the halogen substituents and on their capability as HaB donors, and on the nucleophilic properties of the polysaccharide‐based selector as HaB acceptor. By using carbon dioxide/2‐PrOH and carbon dioxide/MeOH mixtures with different contents of alcoholic co‐solvent, retention and separation factors increased following the order Cl < Br < I in all cases. Compared to *n*‐hexane‐based mobile phases used in HPLC, higher retention factors but lower selectivity values were obtained in SFC in all the studied cases. On the basis of the experimental results, carbon dioxide appeared to work as an interacting solvent compared to the noninteracting *n*‐hexane used in HPLC. Given the capability of carbon dioxide as HaB acceptor and the properties of iodine atoms as powerful HaB donors, QM calculations and MD simulations were performed to confirm that O═C═O^…^I noncovalent interactions could contribute to the huge affinity of the iodinated analyte toward the CDMPC. The computational QM analysis confirmed that only the iodinated analyte **1** can form HaBs with the carbon dioxide oxygen atoms which, differently, form other types of interactions with the brominated and chlorinated analytes **2** and **3**. Furthermore, the MD simulations showed that with a content of 30% of 2‐PrOH in the carbon dioxide‐based mobile phase, the solvation shell around the analyte/selector complexes is formed by 2‐PrOH and carbon dioxide molecules. By using carbon dioxide as virtual solvent, molecules of this solvent were found to participate in the solvation shell of the analyte/chiral selector complexes, also interacting with the CDMPC polymers by HBs. The results of this experimental/computational study suggest that the presence of carbon dioxide may increase the elution times of the iodinated analyte by forming HaB‐based clusters which slow the advancement of the analyte along the column. On the other hand, by using mobile phases containing carbon dioxide, the oxygen atoms of this solvent can coordinate CDMPC through the amidic hydrogen atoms, functioning as an activator of the carbonyl oxygen atoms of the chiral selector as HaB acceptors, thus increasing the affinity of the CDMPC toward HaB donors.

## Conflicts of Interest

The authors declare no conflicts of interest.

## Supporting information




**Supporting File 1**: elps8156‐sup‐0001‐SuppMat.pdf

## Data Availability

Data available in article Supporting Information.

## References

[1] P. Peluso and B. Chankvetadze , “Recognition in the Domain of Molecular Chirality: From Noncovalent Interactions to Separation of Enantiomers,” Chemical Reviews 122 (2022): 13235–13400. 35917234 10.1021/acs.chemrev.1c00846

[2] R. Sardella , F. Ianni , L. Cossignani , G. Aldini , and A. Carotti , “Binding Modes Identification Through Molecular Dynamic Simulations: A Case Study With Carnosine Enantiomers and the Teicoplanin A2‐2‐Based Chiral Stationary Phase,” Journal of Separation Science 43 (2020): 1728–1736. 32112671 10.1002/jssc.202000092

[3] P. Peluso and B. Chankvetadze , “The Molecular Bases of Chiral Recognition in 2‐(Benzylsulfinyl)Benzamide Enantioseparation,” Analytica Chimica Acta 1141 (2021): 194–205. 33248652 10.1016/j.aca.2020.10.050

[4] G. K. E. Scriba , “Update on Chiral Recognition Mechanisms in Separation Science,” Journal of Separation Science 47 (2024): 2400148. 10.1002/jssc.202400148 PMC12964351 38772711

[5] B. Chankvetadze , “Recent Developments on Polysaccharide‐Based Chiral Stationary Phases for Liquid‐Phase Separation of Enantiomers,” Journal of Chromatography A 1269 (2012): 26–51. 23141986 10.1016/j.chroma.2012.10.033

[6] I. Matarashvili , D. Ghughunishvili , L. Chankvetadze , et al., “Separation of Enantiomers of Chiral Weak Acids With Polysaccharide‐Based Chiral Columns and Aqueous‐Organic Mobile Phases in High‐Performance Liquid Chromatography: Typical Reversed‐Phase Behavior?,” Journal of Chromatography A 1483 (2017): 86–92. 28040267 10.1016/j.chroma.2016.12.064

[7] P. Peluso , V. Mamane , R. Dallocchio , A. Dessì , and S. Cossu , “Noncovalent Interactions in High‐Performance Liquid Chromatography Enantioseparations on Polysaccharide‐Based Chiral Selectors,” Journal of Chromatography A 1623 (2020): 461202. 32505290 10.1016/j.chroma.2020.461202

[8] P. Peluso and B Chankvetadze , “Fundamentals of Enantioselective Liquid Chromatography,” in Liquid Chromatography: Fundamentals and Instrumentation, 3rd ed. ed. S. Fanali , B. Chankvetadze , P. R. Haddad , C. F. Poole , and M.‐L. Riekkola (Elsevier, 2023), 383–439.

[9] B. Chankvetadze , E. Yashima , and Y. Okamoto , “Dimethyl‐, Dichloro‐ and Chloromethylphenylcarbamates of Amylose as Chiral Stationary Phases for High‐Performance Liquid Chromatography,” Journal of Chromatography A 694 (1995): 101–109.

[10] R. Dallocchio , B. Sechi , A. Dessì , et al., “Enantioseparations of Polyhalogenated 4,4′‐Bipyridines on Polysaccharide‐Based Chiral Stationary Phases and Molecular Dynamics Simulations of Selector‐Selectand Interactions,” Electrophoresis 42 (2021): 1853–1863. 33742705 10.1002/elps.202100049

[11] R. Dallocchio , A. Dessì , B. Sechi , et al., “Enantioseparation of Planar Chiral Ferrocenes on Cellulose‐Based Chiral Stationary Phases: Benzoate Versus Carbamate Pendant Groups,” Electrophoresis 44 (2023): 203–216. 36177685 10.1002/elps.202200205

[12] P. Wendt , J. Bader , P. L. Waltersmann , et al., “Halogen Bonding in *N*‐Alkyl‐Bromo‐/Iodo‐Pyridinium Salts and Its Application in Chromatography,” Chemistry: A European Journal 30 (2024): e202403062. 39316035 10.1002/chem.202403062

[13] P. Peluso , A. Dessì , R. Dallocchio , et al., “Enantioseparation of 5,5′‐Dibromo‐2,2′‐Dichloro‐3‐Selanyl‐4,4′‐Bipyridines on Polysaccharide‐Based Chiral Stationary Phases: Exploring Chalcogen Bonds in Liquid‐Phase Chromatography,” Molecules (Basel, Switzerland) 26 (2021): 221. 33406753 10.3390/molecules26010221 PMC7794968

[14] G. Kobidze , G. Sprega , A. F. Lo Faro , A. Belloni , P. Peluso , T. Farkas , et al., “Insights Into Separation, Enantioseparation and Recognition Mechanisms of Methamphetamine Isotopologues on Achiral and Polysaccharide‐Based Chiral Columns in High‐Performance Liquid Chromatography,” Analytica Chimica Acta 1337 (2025): 343542. 39800501 10.1016/j.aca.2024.343542

[15] P. Peluso , V. Mamane , R. Dallocchio , et al., “Polysaccharide‐Based Chiral Stationary Phases as Halogen Bond Acceptors: A Novel Strategy for Detection of Stereoselective σ‐Hole Bonds in Solution,” Journal of Separation Science 41 (2018): 1247–1256. 29239526 10.1002/jssc.201701206

[16] P. Peluso , A. Dessì , R. Dallocchio , V. Mamane , and S. Cossu , “Recent Studies of Docking and Molecular Dynamics Simulation for Liquid‐Phase Enantioseparations,” Electrophoresis 40 (2019): 1881–1896. 30710444 10.1002/elps.201800493

[17] R. Sardella , E. Camaioni , A. Macchiarulo , A. Gioiello , M. Marinozzi , and A. Carotti , “Computational Studies in Enantioselective Liquid Chromatography: Forty Years of Evolution in Docking‐ and Molecular Dynamics‐Based Simulations,” Trends in Analytical Chemistry 122 (2020): 115703.

[18] R. Sardella , F. Ianni , A. Macchiarulo , L. Pucciarini , A. Carotti , and B. Natalini , “Elucidation of the Chromatographic Enantiomer Elution Order Through Computational Studies,” Mini‐Reviews in Medicinal Chemistry 18 (2018): 88–97. 27758684 10.2174/1389557516666161018143629

[19] R. Dallocchio , A. Dessì , B. Sechi , and P. Peluso , “Molecular Dynamics Simulations of Amylose‐ and Cellulose‐Based Selectors and Related Enantioseparations in Liquid Phase Chromatography,” Molecules (Basel, Switzerland) 28 (2023): 7419. 37959839 10.3390/molecules28217419 PMC10647714

[20] G. R. Desiraju , P. S. Ho , L. Kloo , et al., “Definition of the Halogen Bond (IUPAC Recommendations 2013),” Pure and Applied Chemistry 85 (2013): 1711–1713.

[21] P. R. Varadwaj , H. M. Marques , A. Varadwaj , and K. Yamashita , “Definition of the Halogen Bond (IUPAC Recommendations 2013): A Revisit,” Crystal Growth & Design 24 (2024): 5494–5525.

[22] P. R. Varadwaj , H. M. Marques , A. Varadwaj , and K. Yamashita , “π‑Hole Halogen Bonds Are Sister Interactions to σ‑Hole Halogen Bonds,” Crystal Growth & Design 24 (2024): 7789–7807.

[23] G. Cavallo , P. Metrangolo , R. Milani , et al., “The Halogen Bond,” Chemical Reviews 116 (2016): 2478–2601. 26812185 10.1021/acs.chemrev.5b00484 PMC4768247

[24] R. Tepper and U. S. Schubert , “Halogen Bonding in Solution: Anion Recognition, Templated Self‐Assembly, and Organocatalysis,” Angewandte Chemie International Edition 57 (2018): 6004–6016. 29341377 10.1002/anie.201707986

[25] K. Mu , Z. Zhu , A. Abula , C. Peng , W. Zhu , and Z. Xu , “Halogen Bonds Exist Between Noncovalent Ligands and Natural Nucleic Acids,” Journal of Medicinal Chemistry 65 (2022): 4424–4435. 35276046 10.1021/acs.jmedchem.1c01854

[26] P. Peluso , V. Mamane , A. Dessì , R. Dallocchio , E. Aubert , C. Gatti , et al., “Halogen Bond in Separation Science: A Critical Analysis Across Experimental and Theoretical Results,” Journal of Chromatography A 1616 (2020): 460788. 31866134 10.1016/j.chroma.2019.460788

[27] E. Kanao , T. Morinaga , T. Kubo , et al., “Separation of Halogenated Benzenes Enabled by Investigation of Halogen–π Interactions With Carbon Materials,” Chemical Science 11 (2020): 409–418. 32190261 10.1039/c9sc04906a PMC7067276

[28] P. Peluso , V. Mamane , E. Aubert , and S. Cossu , “Insights Into the Impact of Shape and Electronic Properties on the Enantioseparation of Polyhalogenated 4,4′‐Bipyridines on Polysaccharide‐Type Selectors. Evidence of Stereoselective Halogen Bonding Interactions,” Journal of Chromatography A 1345 (2014): 182–192. 24792693 10.1016/j.chroma.2014.04.040

[29] P. Peluso , V. Mamane , E. Aubert , A. Dessì , R. Dallocchio , and A. Dore , “Insights Into Halogen Bond‐Driven Enantioseparations,” Journal of Chromatography A 1467 (2016): 228–238. 27328882 10.1016/j.chroma.2016.06.007

[30] D. Speybrouck and E. Lipka , “Preparative Supercritical Fluid Chromatography: A Powerful Tool for Chiral Separations,” Journal of Chromatography A 1467 (2016): 33–55. 27524302 10.1016/j.chroma.2016.07.050

[31] C. West , “Supercritical Fluid Chromatography Is Not (only) Normal‐Phase Chromatography,” Journal of Chromatography A 1713 (2024): 464546. 38041976 10.1016/j.chroma.2023.464546

[32] P. Raveendran , Y. Ikushima , and S. L. Wallen , “Polar Attributes of Supercritical Carbon Dioxide,” Accounts of Chemical Research 38 (2005): 478–485. 15966714 10.1021/ar040082m

[33] X. Zhu , Y. Lu , C. Peng , J. Hu , H. Liu , and Y. Hu , “Halogen Bonding Interactions Between Brominated Ion Pairs and CO_2_ Molecules: Implications for Design of New and Efficient Ionic Liquids for CO_2_ Absorption,” Journal of Physical Chemistry B 115 (2011): 3949–3958. 21413740 10.1021/jp111194k

[34] L. H. Ying , Y. X. Lu , X. Zhu , et al., “CO_2_ Capture Through Halogen Bonding: A Theoretical Perspective,” Science China Chemistry 55 (2012): 1566–1572.

[35] GaussView 6 , R. Dennington , T. A. Keith , and J. M Millam , (Semichem Inc., 2016).

[36] Gaussian 16W, Revision C.01 , M. J. Frisch , G. W. Trucks , H. B. Schlegel , G. E. Scuseria , M. A. Robb , and J. R. Cheeseman , et al., (Gaussian, Inc., 2016).

[37] S. F. Boys and F. Bernardi , “The Calculation of Small Molecular Interactions by the Differences of Separate Total Energies. Some Procedures With Reduced Errors,” Molecular Physics 19 (1970): 553–566.

[38] A. Bondi , “van der Waals Volume and Radii,” Journal of Physical Chemistry 68 (1964): 441–451.

[39] D. A. Case , H. M. Aktulga , K. Belfon , et al., AMBER 2024 (University of California, 2024).

[40] E. F. Pettersen , T. D. Goddard , C. C. Huang , et al., “UCSF Chimera—A Visualization System for Exploratory Research and Analysis,” Journal of Computational Chemistry 25 (2004): 1605–1612. 15264254 10.1002/jcc.20084

[41] V. Mamane , P. Peluso , E. Aubert , S. Cossu , and P. Pale , “Chiral Hexahalogenated 4,4′‐Bipyridines,” Journal of Organic Chemistry 81 (2016): 4576–4587. 27149320 10.1021/acs.joc.6b00413

[42] S. Khater , M. A. Lozac'h , I. Adam , E. Francotte , and C. West , “Comparison of Liquid and Supercritical Fluid Chromatography Mobile Phases for Enantioselective Separations on Polysaccharide Stationary Phases,” Journal of Chromatography A 1467 (2016): 463–472. 27378250 10.1016/j.chroma.2016.06.060

[43] J. R. Strubinger , H. Song , and J. F. Parcher , “High‐Pressure Phase Distribution Isotherms for Supercritical Fluid Chromatographic Systems. 2. Binary Isotherms of Carbon Dioxide and Methanol,” Analytical Chemistry 63 (1991): 104–108.

[44] N. Bargmann‐Leyder , A. Tambuté , and M. Caude , “A Comparison of LC and SFC for Cellulose‐ and Amylose‐Derived Chiral Stationary Phases,” Chirality 7 (1995): 311–325.

[45] B. Chankvetadze , C. Yamamoto , and Y. Okamoto , “Enantioseparation of Selected Chiral Sulfoxides Using Polysaccharide‐Type Chiral Stationary Phases and Polar Organic, Polar Aqueous–Organic and Normal‐Phase Eluents,” Journal of Chromatography A 922 (2001): 127–137. 11486857 10.1016/s0021-9673(01)00958-x

[46] F. London , “The General Theory of Molecular Forces,” Transactions of the Faraday Society 33 (1937): 8–26.

[47] P. Peluso , V. Mamane , E. Aubert , and S. Cossu , “High‐Performance Liquid Chromatography Enantioseparation of Atropisomeric 4,4′‐Bipyridines on Polysaccharide‐Type Chiral Stationary Phases: Impact of Substituents and Electronic Properties,” Journal of Chromatography A 1251 (2012): 91–100. 22771066 10.1016/j.chroma.2012.06.035

[48] J. A. Maier , C. Martinez , K. Kasavajhala , L. Wickstrom , K. E. Hauser , and C. Simmerling , “ff14SB: Improving the Accuracy of Protein Side Chain and Backbone Parameters From ff99SB,” Journal of Chemical Theory and Computation 11 (2015): 3696–3713. 26574453 10.1021/acs.jctc.5b00255 PMC4821407

[49] M. A. A. Ibrahim , “Molecular Mechanical Perspective on Halogen Bonding,” Journal of Molecular Modeling 18 (2012): 4625–4638. 22643975 10.1007/s00894-012-1454-8

[50] M. Kolář , P. Hobza , and K. Bronowska , “Plugging the Explicit σ‐Holes in Molecular Docking,” Chemical Communications 49 (2013): 981–983. 23257988 10.1039/c2cc37584b

[51] R. Dallocchio , A. Dessì , M. Solinas , A. Arras , S. Cossu , and E. Aubert , “Halogen Bond in High‐Performance Liquid Chromatography Enantioseparations: Description, Features and Modelling,” Journal of Chromatography A 1563 (2018): 71–81. 29871805 10.1016/j.chroma.2018.05.061

