# Supplementary material for: Insights Into the Enantioseparation of Polyhalogenated 4,4′‐Bipyridines With a Cellulose Tris(3,5‐Dimethylphenylcarbamate)‐Based Chiral Column by Using Supercritical Fluid Chromatography
Source: Electrophoresis. 2025 May 31;46(11-12):702–15. doi: 10.1002/elps.8156 (PMC12366248; doi:10.1002/elps.8156)
Supplement: Supplementary file 1 — Supporting File 1: elps8156‐sup‐0001‐SuppMat.pdf [file ELPS-46--s001.pdf]

Supporting Information

Insights into the enantioseparation of polyhalogenated 4,4'-bipyridines with a cellulose *tris*(3,5-dimethylphenylcarbamate)-based chiral column by using supercritical fluid chromatography

Emmanuelle Lipka,[1] Roberto Dallocchio,[2] Barbara Sechi,[2] Mikheil Rukhaia,[3] Giorgi Jibuti,[4] Bezhan Chankvetadze,[4] Victor Mamane,[5]* and Paola Peluso[2]*

[1] Laboratoire de Chimie Analytique - Faculté de Pharmacie de Lille, BP 83 – 59006 Lille Cedex, France.

[2] Institute of Biomolecular Chemistry ICB, CNR, Li Punti, Sassari 07100, Italy

[3] Institute of Applied Mathematics, Tbilisi State University, Tbilisi, Georgia

[4] Institute of Physical and Analytical Chemistry, School of Exact and Natural Sciences, Tbilisi State University, Tbilisi 0179, Georgia

[5] Institut de Chimie de Strasbourg, UMR CNRS 7177, Equipe CLIC, 67008, Strasbourg Cedex, France

Table of contents

S1. Additional HPLC and SFC data pag. 2

S2. Additional QM data 3

S3. Molecular dynamics (MD): additional details 7

S1. Additional HPLC and SFC data

Table S1. Selectivity factors (α) of 4,4'-bipyridines **1-3** on Lux Cellulose-1 [cellulose *tris*(3,5-dimethylphenylcarbamate), CDMPC] with *n*-hexane/2-PrOH 90:10 v/v and *n*-hexane/2-PrOH/MeOH 90:5:5 v/v/v as mobile phases (flow rate = 0.8 ml·min^{-1}, T = 25 °C). [a)]

**4,4'-Bipyridine**	**CDMPC**	**CDMPC**
	n-hexane/2-PrOH 90:10	*n*-hexane/2-PrOH/MeOH 90:5:5
**1**	2.68	1.67
**2**	1.61	1.22
**3**	1.16	1.00

[a)] Data from Peluso P, Mamane V, Dallocchio R, Dessì A, Villano R, Sanna D, Aubert E, Pale P, Cossu S. Polysaccharide-based chiral stationary phases as halogen bond acceptors: a novel strategy for detection of stereoselective σ-hole bonds in solution. J Sep Sci. 2018;41:1247–56.

Table S2. Retention times (*t*), retention factors (*k*) and selectivity factors (α) of 4,4'-bipyridines **1-3** on Lux Cellulose-1 [cellulose *tris*(3,5-dimethylphenylcarbamate)] with carbon dioxide/2-PrOH mixtures as mobile phases (flow rate = 1.5 ml·min^{-1}, T = 40 °C).

**4,4'-Bipyridine**	**2-PrOH%**	t_1 **[min]**	t_2 **[min]**	k_1	k_2	**α**
**1**	50	18.09	34.98	8.09	16.59	2.05
	45	24.35	45.96	11.00	21.64	1.97
	40	34.26	64.93	15.47	30.21	1.95
	30	66.99	126.54	30.60	58.69	1.92
**2**	50	4.42	5.34	1.22	1.68	1.38
	45	4.78	5.77	1.35	1.84	1.36
	40	5.47	6.82	1.63	2.28	1.40
	35	6.45	8.12	2.15	2.96	1.38
	30	8.09	10.5	2.81	3.95	1.40
	20	13.63	18.36	5.14	7.27	1.41
	10	32.42	44.21	12.68	17.65	1.39
**3**	50	3.07	3.07	0.54	0.54	1
	40	3.24	3.24	0.56	0.56	1
	30	3.76	3.86	0.77	0.82	1.06
	20	4.98	5.13	1.24	1.31	1.06
	10	7.62	7.96	2.22	2.36	1.06

Table S3. Retention times (*t*), retention factors (*k*) and selectivity factors (α) of 4,4'-bipyridines **1-3** on Lux Cellulose-1 [cellulose *tris*(3,5-dimethylphenylcarbamate)] with *n*-hexane/2-PrOH mixtures as mobile phases (flow rate = 0.8 ml·min^{-1}, T = 22 °C).

**4,4'-Bipyridine**	**2-PrOH%**	t_1 **[min]**	t_2 **[min]**	k_1	k_2	**α**
**1**	50	14.30	32.94	2.76	7.70	2.77
	40	16.42	39.34	3.41	9.57	2.81
	30	19.92	48.57	4.46	12.32	2.76
	20	26.57	68.17	6.28	17.69	2.82
**2**	50	7.09	9.03	0.87	1.38	1.59
	40	7.17	9.25	0.93	1.49	1.61
	30	7.32	9.52	1.01	1.61	1.60
	20	7.82	10.49	1.14	1.87	1.64
**3**	50	5.22	5.39	0.38	0.42	1.12
	40	5.07	5.21	0.36	0.40	1.10
	30	5.09	5.22	0.40	0.43	1.10
	20	5.23	5.54	0.43	0.52	1.20

S2. Additional QM data

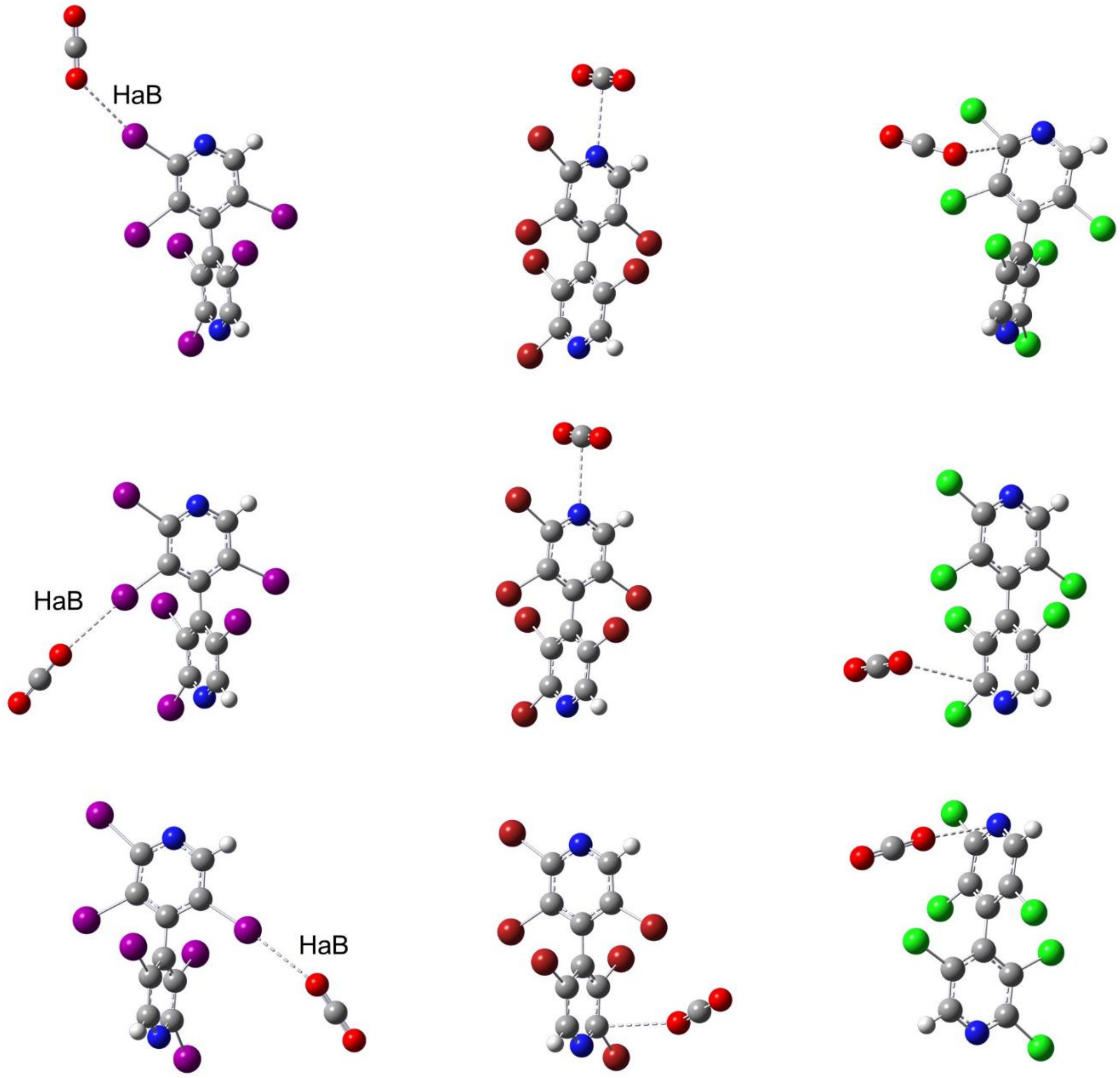

Figure S1. DFT optimized complexes between 4,4'-bipyridines **1**-**3** and CO_2 (color legend: bromine, dark red; carbon, grey; chlorine, green; hydrogen, pale grey; iodine, magenta; nitrogen, blue; oxygen, red) (energies and geometrical parameters of noncovalent interactions are reported in Table 2, main text).

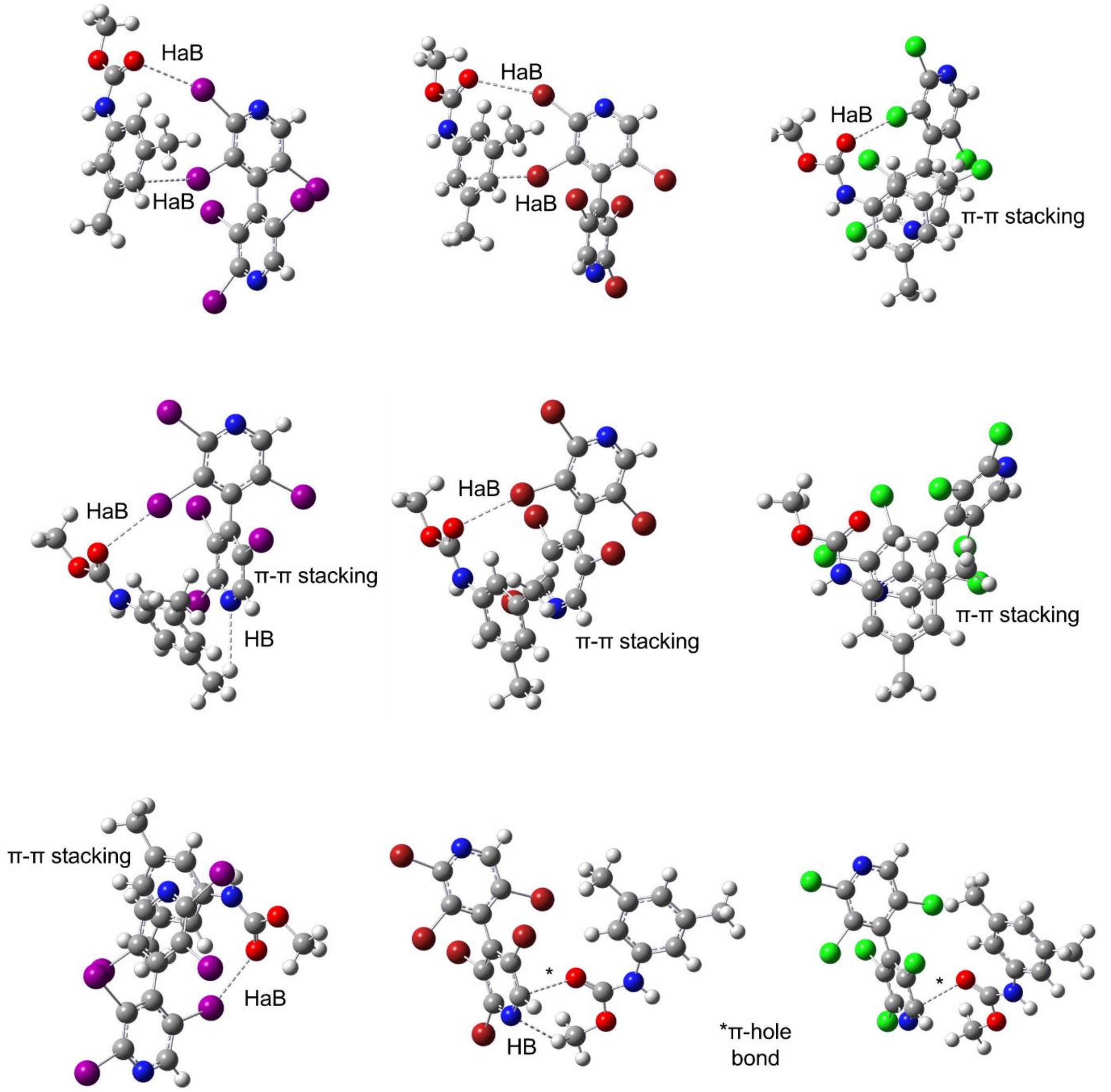

Figure S2. DFT optimized complexes between 4,4'-bipyridines **1**-**3** and MDMPC (input file: carbonyl group pointing toward the analytes) (color legend: bromine, dark red; carbon, grey; chlorine, green; hydrogen, pale grey; iodine, magenta; nitrogen, blue; oxygen, red) (energies and geometrical parameters of noncovalent interactions are reported in Table 3, main text).

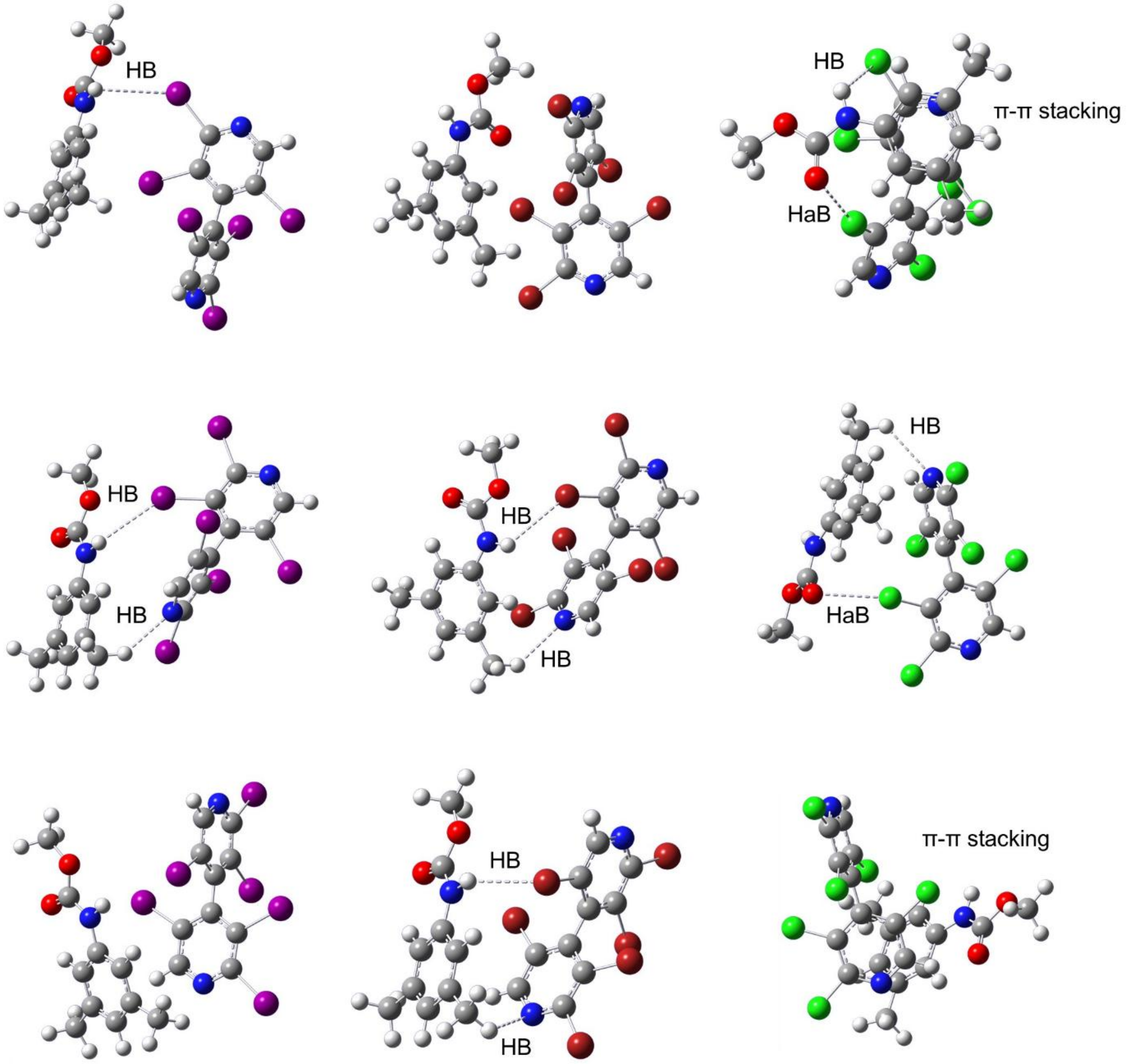

Figure S3. DFT optimized complexes between 4,4'-bipyridines **1**-**3** and MDMPC (input file: N-H group pointing toward the analytes) (color legend: bromine, dark red; carbon, grey; chlorine, green; hydrogen, pale grey; iodine, magenta; nitrogen, blue; oxygen, red) (energies and geometrical parameters of noncovalent interactions are reported in Table 3, main text).

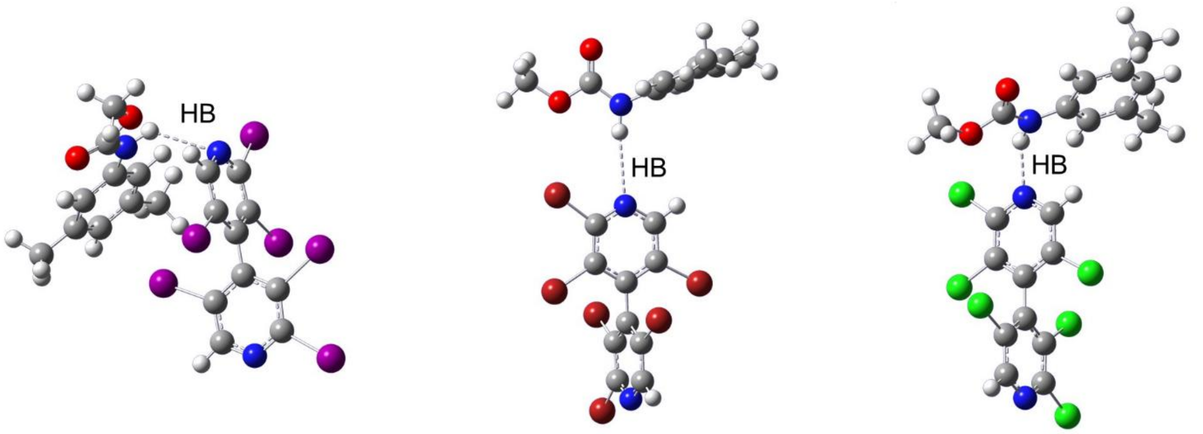

Figure S4. DFT optimized complexes between 4,4'-bipyridines **1-3** and MDMPC (input file: N-H group pointing toward the N_{pyr} of the analytes) (color legend: bromine, dark red; carbon, grey; chlorine, green; hydrogen, pale grey; iodine, magenta; nitrogen, blue; oxygen, red) (energies and geometrical parameters of noncovalent interactions are reported in Table 3, main text).

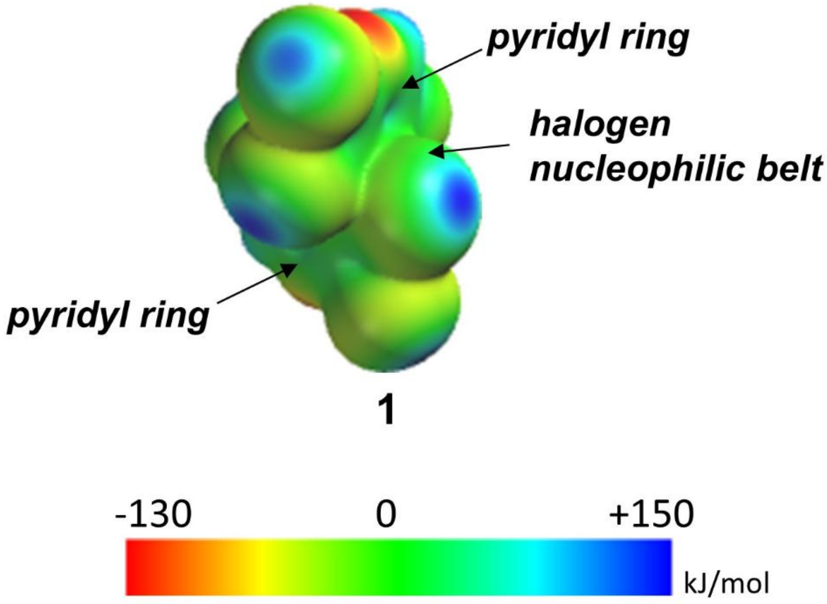

Figure S5. Electrostatic potential mapped on the electro density isosurface computed for the DFT optimized structure of (*P*)-**1**.

S3. Molecular dynamics (MD): additional details

MD: detailed description

For the MD simulations, 4,4'-bipyridine **1** were constructed as described in section 2.1 Quantum mechanics (main text). The explicit σ-hole (ESH) was used, as previously described [1-3], to account for charge anisotropy of the electrostatic potential on top of the iodine atoms. On this basis, a massless dummy atom connected to the iodine atoms was introduced manually, by using distance and charge values as described by Hobza and co-authors [2]. The parameters used for I were 1.6 Å, and 0.3 units of positive charge for the extra point (ExP), respectively (Table S4).

Table S4. Parameters used for the ExP of charge (X = I) [1,2].

**Mass ExP**	**0.00 amu**
r (EP)	1.00 Å
ε (EP)	0.00 Å
r_{eq} (I-EP)	1.60 Å
K_r (X-EP)	600.0
Θ_{eq} (A-I-EP)	180.0°
K_θ (A-I-EP)	150.0
γ (A-A-I-EP)	0.00°
V_n (A-A-I-EP)	0.00

The AMBER24 Antechamber toolkit (University of California, San Francisco, USA) was used to assign the generalized Amber Force Field (GAFF) atom type and the AM1-BCC type of charge to 4,4'-bipyridine **1**. RESP charges were computed for carbon dioxide. A nonamer of cellulose *tris*(3,5-dimethylphenylcarbamate (CDMPC) was used as virtual polymer, which was prepared and optimized as previously reported [3]. The AMBER24 software was used to carry out the MD simulations. *n*-Hexane/2-PrOH 70:30, carbon dioxide/2-PrOH 70:30, or pure carbon dioxide solvation effects were considered by means of the explicit periodic solvent box (TIP3P). Periodic boundary conditions were applied to simulate a continuous system. The overall MD process consisted of four steps. The first two steps aimed to relax the system and search for local minimal conformation energies. The system was submitted to a series of 13 alternating minimization and dynamic steps. At the beginning, only hydrogen and solvent were free to move, and finally all atoms were released to move freely, except the terminal methoxyl groups of the polymer chain. For each step, energy minimization was executed by the steepest descent method over 3000 steps and the conjugate gradient method for the subsequent 7000 steps. The integration time step was of 2 fs, and non-bonded cutoff of 10 Å, (8 Å in the production run). Then, the third equilibrating

step consisted of a gradual temperature increase. In the equilibration step, after a first dynamics using the NVT ensemble of 0.5 ns at 300 K, four steps of dynamics using NPT ensemble was executed, ranging from 0.06 to 0.2 ns, with heating ramp from 100 K until 300 K. Finally, the production step was carried out under the equilibrium conditions and the system was subjected to 100 ns MD simulation for each enantiomer of **1** on the CDMPC nonamer. The Chimera software (UCSF, San Francisco, USA) was used for visualization and analysis of the MD trajectories.

[1] Ibrahim MAA. Molecular mechanical perspective on halogen bonding. J Mol Model. 2012;18:4625-38.

[2] Kolar M, Hobza P, Bronowska K. Plugging the explicit σ-holes in molecular docking. Chem Commun. 2013;49:981-3.

[3] Dallocchio R, Dessì A, Solinas M, Arras A, Cossu S, Aubert E, Mamane V, Peluso P. Halogen bond in high-performance liquid chromatography enantioseparations: description, features and modelling. J Chromatogr A. 2018;1563:71–81.

Table S5. Noncovalent interactions (NCIs) and related lifetime observed in 100 ns MD of the (*M*)-**1**/CDMPC complex in *n*-hexane/2-PrOH 70:30 solvent box.

**Type**	**Acceptor**	**Donor**	**Contact**	**NCI**	**Lifetime (ns)** **max**	**min**
Solvent-Solvent	2-PrOH	2-PrOH	2-PrOH···O(H)CHMe$_2$	HB	7	0.02
CDMPC-CDMPC	CDMPC	CDMPC	>C=O···H-N<	HB	49	0.02
Solvent-CDMPC	CDMPC	2-PrOH	2-PrOH···O	HB	34	0.02
	2-PrOH	CDMPC	2-Pr(H)O···H-N<	HB	25	0.02
Solvent-Analyte	Analyte	2-PrOH	2-PrOH···N_{pyr}	HB	0.8	0.02
	2-PrOH	Analyte	2-Pr(H)O···I	HaB	5	0.02
Analyte-CDMPC	CDMPC	Analyte	I···O=C<	HaB	78	43

Table S6. Noncovalent interactions (NCIs) and related lifetime observed in 100 ns MD of the (*P*)-**1**/CDMPC complex in *n*-hexane/2-PrOH 70:30 solvent box.

**Type**	**Acceptor**	**Donor**	**Contact**	**NCI**	**Lifetime (ns)** **max**	**min**
Solvent-Solvent	2-PrOH	2-PrOH	2-PrOH···O(H)CHMe$_2$	HB	7	0.02
CDMPC-CDMPC	CDMPC	CDMPC	>C=O···H-N<	HB	63	0.02
Solvent-CDMPC	CDMPC	2-PrOH	2-PrOH···O	HB	26	0.02
	2-PrOH	CDMPC	2-Pr(H)O···H-N<	HB	17	0.02
Solvent-Analyte	Analyte	2-PrOH	2-PrOH···N_{pyr}	HB	3	0.02
	2-PrOH	Analyte	2-Pr(H)O···I	HaB	10	0.02
Analyte-CDMPC	CDMPC	Analyte	I···O=C<	HaB	70	6
	Analyte	CDMPC	N_{pyr}···H-N<	HB	0.6	0.6

Table S7. Noncovalent interactions (NCIs) and related lifetime observed in 100 ns MD of the (*M*)-**1**/CDMPC complex in CO_2/2-PrOH 70:30 solvent box.

**Type**	**Acceptor**	**Donor**	**Contact**	**NCI**	**Lifetime (ns)** **max**	**min**
Solvent-Solvent	2-PrOH	2-PrOH	2-PrOH···O(H)CHMe$_2$	HB	2	0.02
	CO_2	2-PrOH	2-PrOH···O=C=O	HB	0.1	0.02
CDMPC-CDMPC	CDMPC	CDMPC	>C=O···H-N<	HB	60	0.02
Solvent-CDMPC	CDMPC	2-PrOH	2-PrOH···O	HB	77	0.02
	2-PrOH	CDMPC	2-Pr(H)O···H-N<	HB	49	0.02
	CO_2	CDMPC	O=C=O···H-N<	HB	0.4	0.02
Solvent-Analyte	Analyte	2-PrOH	2-PrOH···N_{pyr}	HB	2	0.02
	2-PrOH	Analyte	2-Pr(H)O···I	HaB	35	0.02
	CO_2	Analyte	O=C=O···I	HaB	0.4	0.02
Analyte-CDMPC	CDMPC	Analyte	I···O=C<	HaB	99	2.8
	Analyte	CDMPC	N_{pyr}···H-N<	HB	0.1	0.1

Table S8. Noncovalent interactions (NCIs) and related lifetime observed in 100 ns MD of the (*P*)-**1**/CDMPC complex in CO_2/2-PrOH 70:30 solvent box.

Type	Acceptor	Donor	Contact	NCI	Lifetime (ns) max	min
Solvent-Solvent	2-PrOH	2-PrOH	2-PrOH···O(H)CHMe$_2$	HB	8	0.02
	CO_2	2-PrOH	2-PrOH···O=C=O	HB	0.2	0.02
CDMPC-CDMPC	CDMPC	CDMPC	>C=O···H-N<	HB	60	0.02
Solvent-CDMPC	CDMPC	2-PrOH	2-PrOH···O	HB	25	0.02
	2-PrOH	CDMPC	2-Pr(H)O···H-N<	HB	16	0.02
	CO_2	CDMPC	O=C=O···H-N<	HB	2	0.02
Solvent-Analyte	Analyte	2-PrOH	2-PrOH···N_{pyr}	HB	0.5	0.02
	2-PrOH	Analyte	2-Pr(H)O···I	HaB	1.4	0.02
	CO_2	Analyte	O=C=O···I	HaB	0.3	0.02
Analyte-CDMPC	CDMPC	Analyte	I···O=C<	HaB	73	18
	Analyte	CDMPC	N_{pyr}···H-N<	HB	9	9

Table S9. Noncovalent interactions (NCIs) and related lifetime observed in 100 ns MD of the (*M*)-**1**/CDMPC complex in CO_2 solvent box.

Type	Acceptor	Donor	Contact	NCI	Lifetime (ns) max	min
CDMPC-CDMPC	CDMPC	CDMPC	>C=O···H-N<	HB	74	0.02
Solvent-CDMPC	CO_2	CDMPC	O=C=O···H-N<	HB	0.4	0.02
Solvent-Analyte	CO_2	Analyte	O=C=O···I	HaB	0.3	0.02
Analyte-CDMPC	CDMPC	Analyte	I···O=C<	HaB	83	15
	Analyte	CDMPC	N_{pyr}···H-N<	HB	0.2	0.2

Table S10. Noncovalent interactions (NCIs) and related lifetime observed in 100 ns MD of the (*P*)-**1**/CDMPC complex in CO_2 solvent box.

Type	Acceptor	Donor	Contact	NCI	Lifetime (ns) max	min
CDMPC-CDMPC	CDMPC	CDMPC	>C=O···H-N<	HB	71	0.02
Solvent-CDMPC	CO_2	CDMPC	O=C=O···H-N<	HB	0.2	0.02
Solvent-Analyte	CO_2	Analyte	O=C=O···I	HaB	0.2	0.02
Analyte-CDMPC	CDMPC	Analyte	I···O=C<	HaB	91	0.4
	Analyte	CDMPC	N_{pyr}···H-N<	HB	0.6	0.6

Table S11. Geometrical parameters of the noncovalent interactions observed in representative snapshots extracted from the molecular dynamic (MD) trajectories of the complexes of (*M*)- and (*P*)-**1** with cellulose *tris*(3,5-dimethylphenylcarbamate) (9-mer CDMPC) with different *n*-hexane/2-PrOH 70:30 (Mix A) and carbon dioxide/2-PrOH 70:30 (Mix B). Representative snapshots of the complexes of (*P*)-**1** with CDMPC are depicted: hexane/2-PrOH 70:30 (A) and carbon dioxide/2-PrOH 70:30 (B).

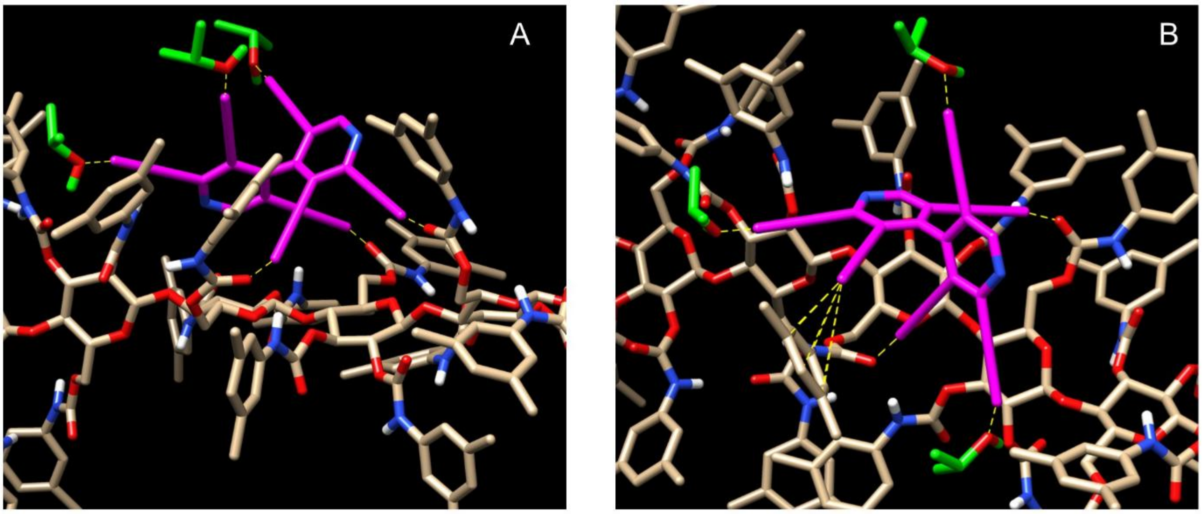

Enant.	Solvent	NCI type[a]	Length (Å)	C-I···O= (°)	I···O=C< (°)	*pp* [b]
(*M*)	Mix A	5-I···O=C<	3.263	164.813	133.723	-6.8%
		2'-I···O=C<	2.987	178.765	145.648	-14.7%
		2-I···O(H)CHMe$_2$	2.655			
		3-I···O(H)CHMe$_2$	3.516			
		3'-I···O(H)CHMe$_2$	3.322			
		5'-I···O(H)CHMe$_2$	3.059			
(*P*)	Mix A	5-I···O=C<	2.942	171.984	135.648	-15.9%
		2'-I···O=C<	3.073	174.529	165.025	-12.2%
		3'-I···O=C<	3.254	166.840	131.768	-7.0%
		2-I···O(H)CHMe$_2$	3.092			
		3-I···O(H)CHMe$_2$	3.145			
		5'-I···O(H)CHMe$_2$	3.157			
(*M*)	Mix B	5-I···O=C<	3.071	177.367	137.786	-12.3%
		2'-I···O=C<	3.008	175.620	134.201	-14.1%
		3-I···π_{ArMe2}	4.155			
		2-I···O(H)CHMe$_2$	3.196			
		3'-I···O(H)CHMe$_2$	3.230			
		5'-I···O(H)CHMe$_2$	3.157			
(*P*)	Mix B	5-I···O=C<	3.097	174.330	139.262	-11.5%
		3'-I···O=C<	3.109	165.031	121.920	-11.2%
		3-I···π_{ArMe2}	4.631			
		5'-I···O(H)CHMe$_2$	3.208			
		2-I···O(H)CHMe$_2$	3.276			
		2'-I···O(H)CHMe$_2$	3.128			

[a] NCI, Noncovalent interaction.
[b] *pp* = penetration parameter: halogen (Ha)···O% = 100 × {($d_{Ha\cdots O}$)/(r_{vdW} Ha + r_{vdW} O) − 1}), where $d_{Ha\cdots O}$ is the interatomic distance and r_{vdW} the corresponding van der Waals radii.

Table S12. Geometrical parameters of the noncovalent interactions observed in a representative snapshot extracted from the molecular dynamic (MD) trajectories of the complexes of (*M*)-**1** (A) and (*P*)-**1** (B) with cellulose *tris*(3,5-dimethylphenylcarbamate) (9-mer CDMPC) with carbon dioxide (solvent box).

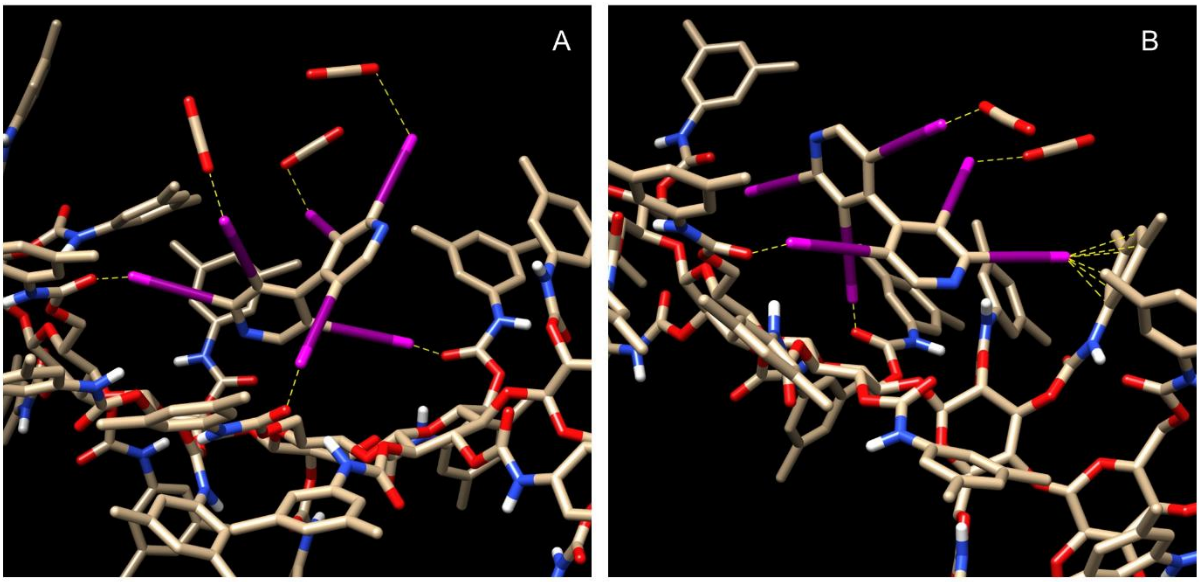

Enant.	NCI type [a]	Length (Å)	C-I···O= (°)	I···O=C< (°)	*pp* [b]
(*M*)	5-I···O=C<	3.210	172.935	135.915	-8.3%
	5'-I···O=C<	3.309	164.247	113.200	-5.5%
	2-I···O=C<	3.126	168.414	147.490	-10.7%
	3-I···O=C=O	3.542			
	3'-I···O=C=O	3.856			
	2-I···O=C=O	4.514			
(*P*)	5-I···O=C<	3.275	169.707	130.670	-6.4%
	3'-I···O=C<	3.033	179.606	113.212	-13.3%
	2-I···π_{ArMe2}	3.513			
	5'-I···O=C=O	3.697			
	3-I···O=C=O	3.836			

[a] NCI, Noncovalent interaction.
[b] *pp* = penetration parameter: halogen (Ha)···O% = 100 × {($d_{Ha\cdots O}$)/(r_{vdW} Ha + r_{vdW} O) − 1}), where $d_{Ha\cdots O}$ is the interatomic distance and r_{vdW} the corresponding van der Waals radii.

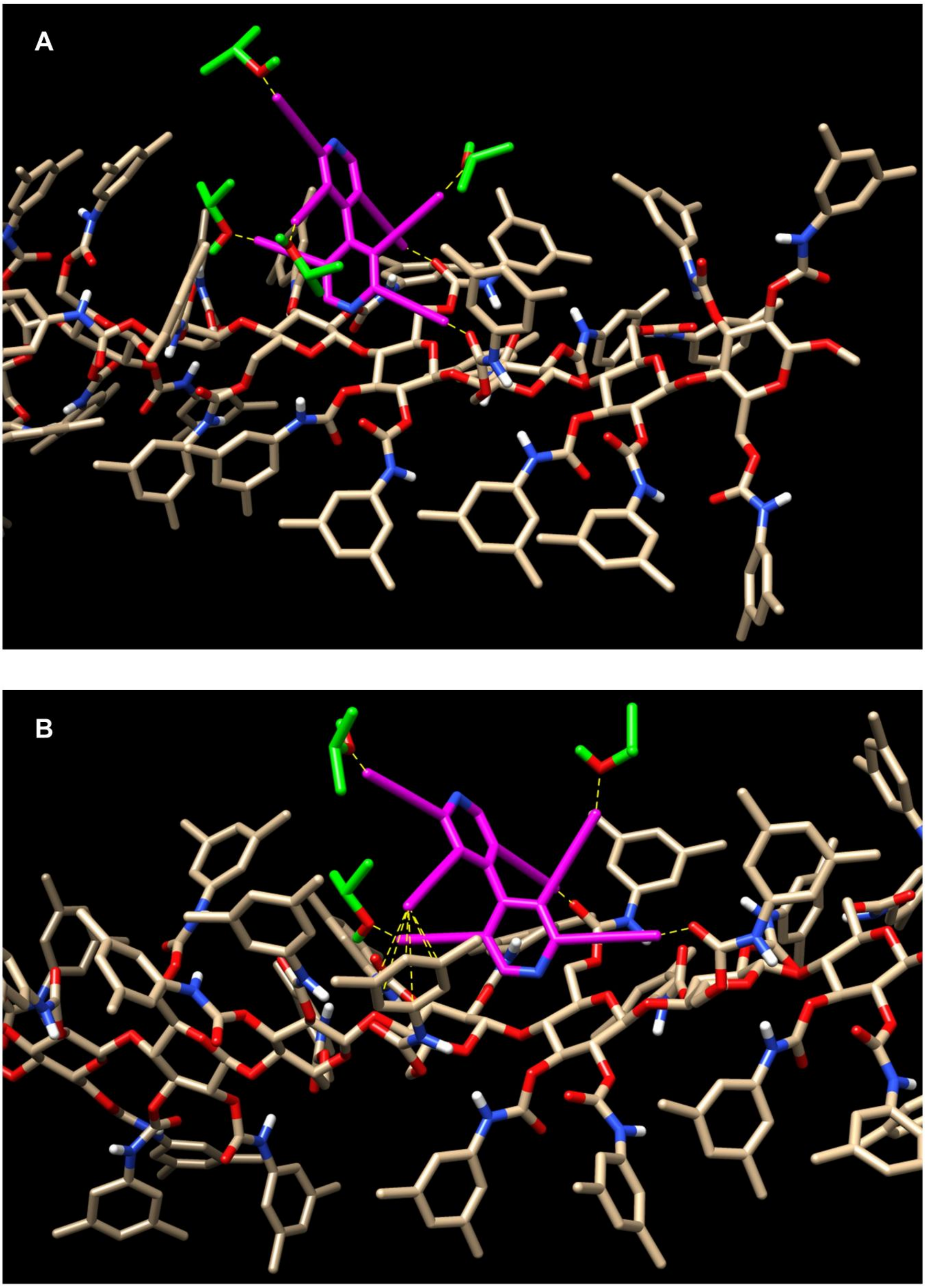

Figure S6. Representative snapshots extracted from the molecular dynamic (MD) trajectories of the complexes of (*M*)-**1** with CDMPC: hexane/2-PrOH 70:30 (A) and carbon dioxide/2-PrOH 70:30 (B).
